# An efficient and green approach for the synthesis of 2,4-dihydropyrano[2,3-*c*]pyrazole-3-carboxylates using Bi_2_O_3_/ZrO_2_ as a reusable catalyst†

**DOI:** 10.1039/c8ra01994k

**Published:** 2018-05-03

**Authors:** Sandeep V. H. S. Bhaskaruni, Suresh Maddila, Werner E. van Zyl, Sreekantha B. Jonnalagadda

**Affiliations:** School of Chemistry & Physics, University of KwaZulu-Natal Westville Campus, Chiltern Hills Durban-4000 South Africa jonnalagaddas@ukzn.ac.za +27 31 2603091 +27 31 2607325

## Abstract

A novel material of bismuth loaded on zirconia (Bi_2_O_3_/ZrO_2_) is synthesized by simple wet-impregnation method and characterized by several techniques (P-XRD, TEM, SEM, BET, *etc.*). Bi_2_O_3_/ZrO_2_ proved to be a good catalyst for the four-component, one-pot reaction to produce a new series of 2,4-dihydropyrano[2,3-*c*]pyrazole-3-carboxylate derivatives with excellent yields (91 to 98%) under mild conditions at RT with short reaction times (≈20 min). The structures of the target molecules were confirmed by ^1^H NMR, ^13^C NMR, ^15^N NMR, HRMS and FT-IR. The catalyst is easily separable and can be reused for six cycles without ostensible loss of activity. This method is inexpensive, atom-efficient and no chromatographic separations are needed.

## Abstract 2

Novel bismuth loaded on zirconia (Bi_2_O_3_/ZrO_2_) as a reusable catalyst to synthesize 2,4-dihydropyrano[2,3-*c*]pyrazole-3-carboxylates under mild conditions.

## Graphical abstract

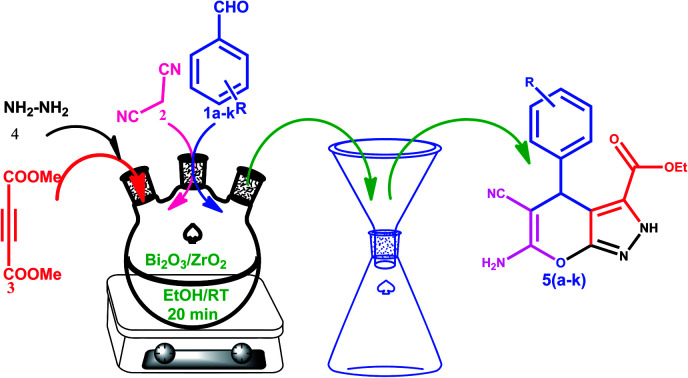

## 1. Introduction

In pharmaceutical research, methods for the synthesis of medicinally important scaffolds in high yields under moderate conditions fascinate all.^1^ Multicomponent reactions (MCRs) are one-step reactions, in which three or more starting materials are integrated together to obtain the target molecule with no need for separation of intermediates.^2^ In MCRs, the product formation takes place through reaction of multiple reactive components present in the reaction media in sequence. The main characteristics are high atom economy, eco-compatibility, and efficient forming of multiple-bonds, which are the near ideal targets in the modern organic synthesis.^3,4^

Heterogeneous catalysts play a key role in the development of cost-effective and eco-friendly protocols in organic synthesis.^5^ The main benefits are the recyclability and reusability of the catalytic material, which are not observed in other organic or inorganic homogenous catalysts.^6^ The principal assets of heterogeneous catalysts are their high surface area, simple handling, low toxicity, short reaction times, easy separation, and thermal and mechanical stability, relative to many homogenous catalysts.^7^

To vary the surface characteristics of heterogeneous catalysts, the use of mixed oxides is an attractive option.^8,9^ The recent literature reveals that zirconium oxide has been used either as an active material or a support in catalysts in the design of various organic transformations, with good product selectivity.^10–12^ ZrO_2_ even shows potential catalyst activity in water. Its redox properties, high surface area, and acidic and basic sites make it superior to other catalytic ESI.†^12^ Furthermore, ZrO_2_ is less-expensive, stable, non-hazardous, reusable and viably available.^3,13^ Bismuth is a green grade element and its related compounds play a prominent role in many organic transformations, such as oxidation, reduction, and C–C bond formation reactions,^14^ owing to the presence of Lewis acidic character. Moreover, it is non-toxic and highly stable.^15^ Hence, the use of bismuth oxide-loaded zirconia catalysts is an elective choice for the present synthetic scheme.

Heterocyclic molecules have become important in the fields of pharmaceutical, agro, industrial and combinatorial chemistry.^16^ Accomplishing facile and easy methods for the design of new composite heterocyclic moieties is a key aspect and ongoing challenge in the field of heterocyclic chemistry. Pyrano[2,3-*c*]pyrazoles and their derivatives are significant nitrogen-containing heterocyclic molecules with interesting biological and pharmaceutical properties, such as anti-inflammatory,^17^ anticancer,^18^ antioxidant,^19^ anti-bacterial^20^ and anti-tubercular agents.^21^ Subsequently, the preparation of several substituted pyrano[2,3-*c*]pyrazole derivatives has been explored by different methods, using silica-supported tetramethylguanidine,^22^ BS-2G-Ti,^23^ Ba(OH)_2_,^24^ γ-alumina,^25^ Amberlyst A21,^26^ acetic acid,^27^ visible light-assisted synthesis^28^*etc.* as catalysts. All these reactions reported have low yields, with many demanding expensive chemicals, harsh reaction conditions and long reaction times. Therefore, an improvement over existing procedures with a greener approach with enhanced yields under milder conditions is necessary and vital.

With consistent interest in development of improved methods for the synthesis of different biologically active scaffolds, we have previously reported varied enriched protocols for the synthesis of novel heterocycles.^29–32^ In this communication, we report a new catalyst material Bi_2_O_3_/ZrO_2_ for MCRs for the synthesis of new functionalized pyrano[2,3-*c*]pyrazole derivatives by using a one-pot four-component reaction.

## 2. Experimental section

### 2.1 Catalyst preparation

A series of bismuth oxide-loaded zirconia (Bi_2_O_3_/ZrO_2_) catalyst materials with different weight percentages were prepared (1, 2.5, & 5 wt%) by employing wet impregnation method.^31–35^ A mixture of zirconium oxide (ZrO_2_, 2 g, Alfa Aesar) and an appropriate amount (wt%) of bismuth chloride (BiCl_3_, Alfa Aesar) in deionised water (60 mL) was agitated with vigorous stirring at room temperature (RT) for 7 h. The resultant slurry was heated to and preserved at 75 °C for 1.5 h and then allowed to cool to RT. Then, the slurry was filtered under vacuum and dried in an oven at 120–140 °C for 8 h, and further calcined at 450 °C for 6 h in the presence of air to afford different wt% of Bi_2_O_3_/ZrO_2_. Instrumentation details are included in the (ESI-I†).

### 2.2 General procedure for the synthesis of pyranopyrazole derivatives (5a–k)

**Scheme 1:**
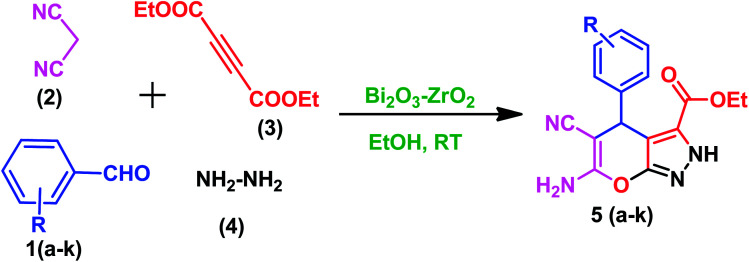
Synthesis of 2,4-dihydropyrano[2,3-*c*] pyrazole-3-carboxylates.

In order to examine the efficiency of the prepared Bi_2_O_3_/ZrO_2_ catalyst, in a 25 mL reaction flask at RT, an equi-molar mixture of the chosen aromatic aldehyde (1 mmol), malononitrile (1 mmol), hydrazine hydrate (1 mmol), diethyl acetylenedicarboxylate (1 mmol) and Bi_2_O_3_/ZrO_2_ (30 mg) catalyst were added under stirring using ethanol as the solvent (5 mL) for 15 minutes (Scheme 1). The progression of the reaction was observed by TLC. After completion of the reaction, the catalyst material was recovered by simple filtration and the organic compound was separated by addition of an appropriate amount of ethanol. Then, the pure target products were obtained after evaporation of ethanol under vacuum. All the reaction products were characterised using various spectral techniques (^1^H-NMR, ^15^N NMR, ^13^C-NMR, HRMS and FT-IR). The related details and spectra are included in the (ESI-II†).

## 3. Results and discussion

### 3.1 XRD analysis

**Fig. 1:**
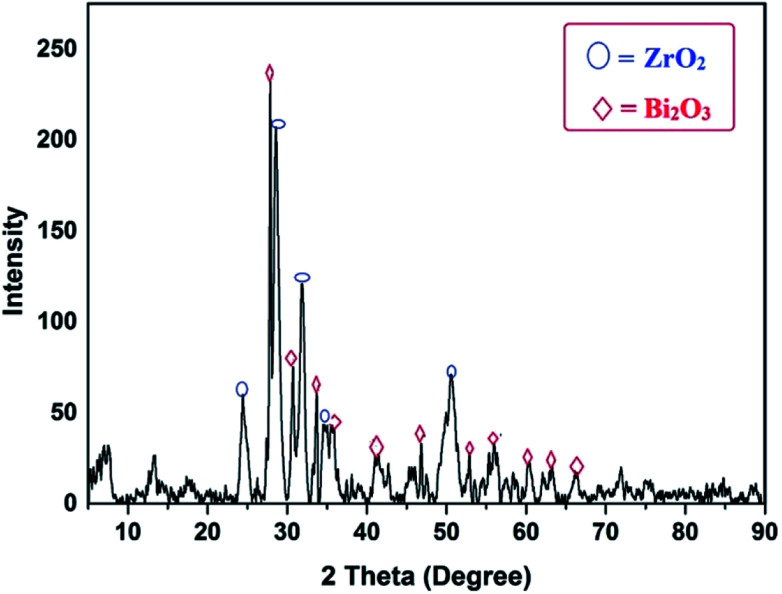
Powder X-ray diffractogram of 2.5% Bi_2_O_3_–ZrO_2_ catalyst.

X-ray diffraction studies were performed to analyze the phases and crystallinity size of the catalyst materials. The powdered XRD patterns of the different wt% of prepared Bi_2_O_3_–ZrO_2_ are shown in Fig. 1 and the diffraction peaks (2 theta) were measured from 0° to 90°. The major diffraction peaks placed at 2*θ* of 24.5°, 27.8°, 31.3°, 35.4° and 50.3° are indexed to the (110), (−111), (111), (200) and (022) diffraction planes of ZrO_2_ and the peaks were also correlated with international standard file (JCPDS 37-1484). The Bi_2_O_3_ peaks were displayed in the XRD diffractogram at 2*θ* = 27.16°, 30.3°, 35.4°,40.3°, 46.9°, 53.4°, 56.1°, 59.4°, 62.9°, 64.5° and 65.9° and furthermore these were matched with (120), (012), (031), (013), (302), (124), (222), (134), (052), (412) and (251) diffraction planes corresponding to the standard file (JCPDS 41-1449). The diffraction pattern reveals the polycrystalline nature of the prepared catalytic material.

### 3.2 TEM analysis

**Fig. 2:**
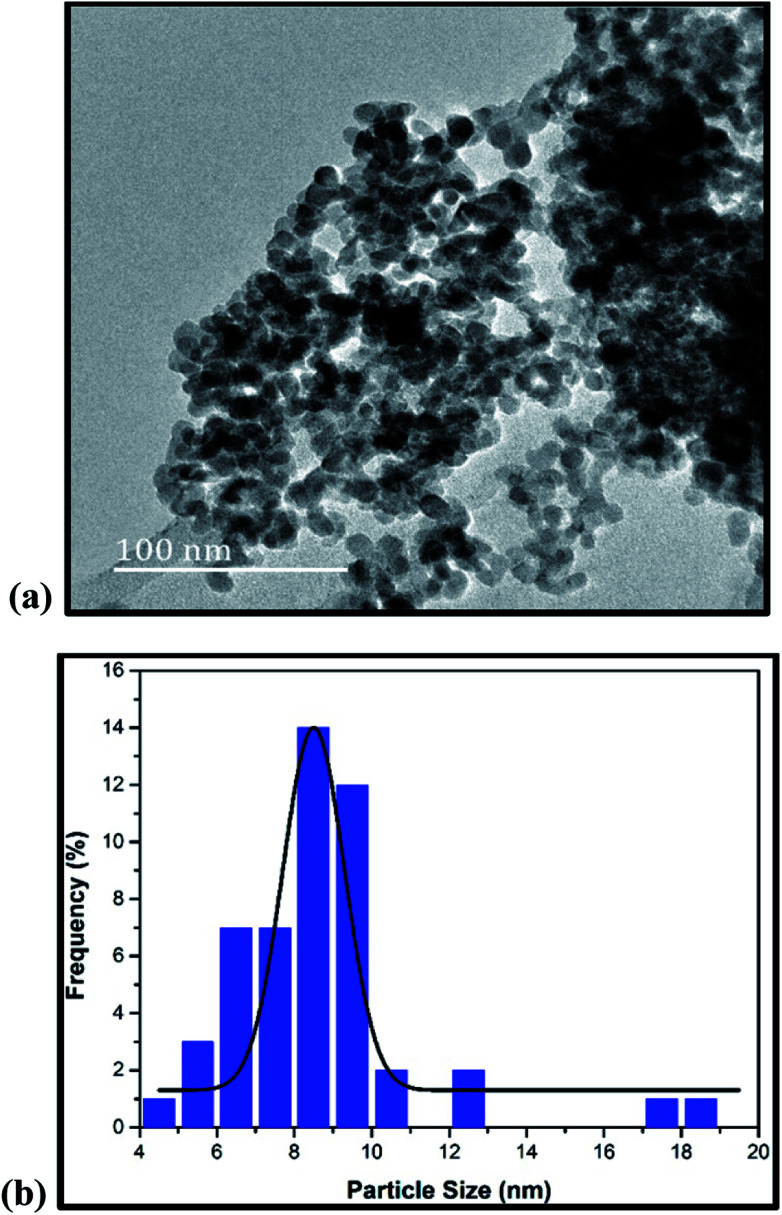
(a) TEM micrograph of 2.5% Bi_2_O_3_/ZrO_2_ catalyst. (b) Particle size distribution of Bi_2_O_3_/ZrO_2_.

The TEM image of 2.5 wt% bismuth loaded on zirconia is shown in Fig. 2a. It shows that bismuth particles settled as irregular black particles on the spherical shaped zirconia particles. The highly dispersed bismuth particles occur due to fine interaction between bismuth and the zirconia oxides. In order to analyse the particle size distribution (Fig. 2b) quantitatively, the histogram was fitted with the Gaussian function and the mean particle size was calculated to be 8.54 nm.

### 3.3 SEM analysis

**Fig. 3:**
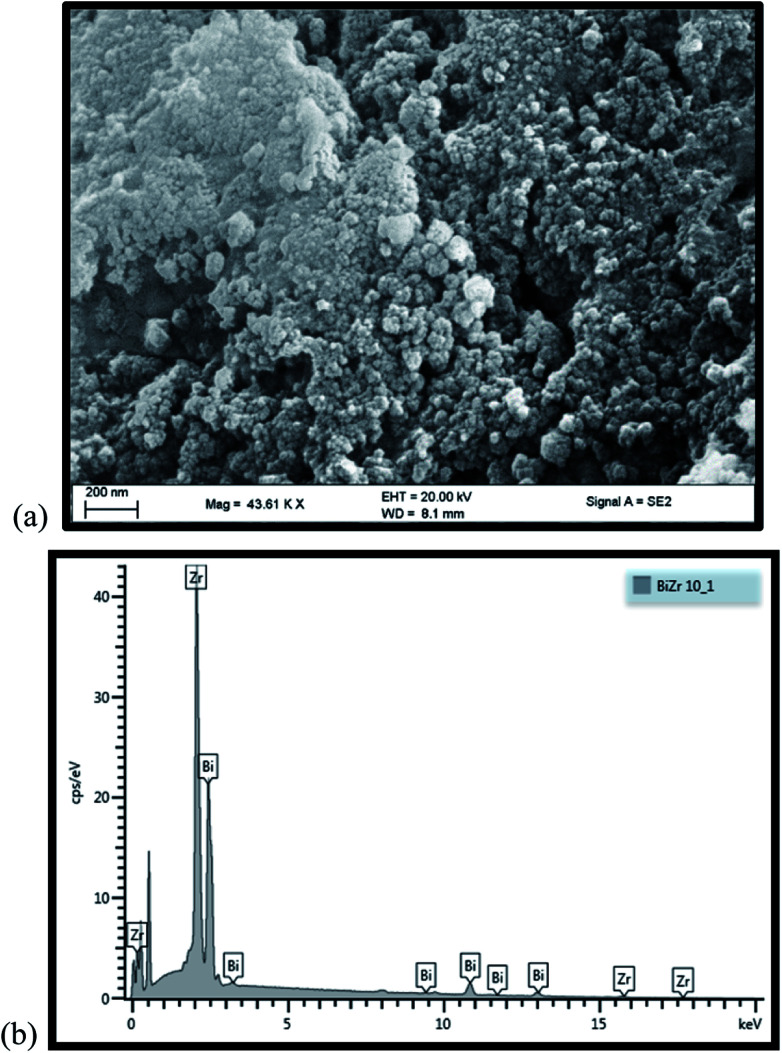
(a) SEM micrograph and (b) EDS spectrum of 2.5% Bi_2_O_3_/ZrO_2_ catalyst.

Fig. 3a displays a scanning electron microscopy (SEM) image of the Bi_2_O_3_/ZrO_2_ combined, which demonstrates the catalyst surface morphology. The units are huge with oval-like irregular shapes. This microgram displays that the Bi_2_O_3_ particles are aggregated and accumulated on the zirconia. A homogeneous distribution of Bi_2_O_3_ on the surface of the ZrO_2_ catalyst was calculated by EDS analysis (Fig. 3b), with minor but prominent quantities of surface improvement of bismuth.

### 3.4 BET surface area analysis

**Fig. 4:**
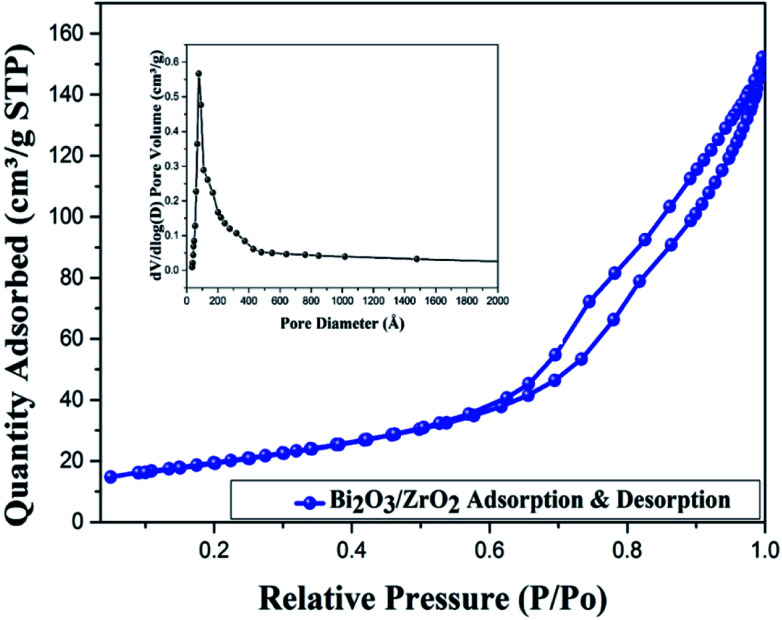
N_2_ adsorption–desorption isotherm of 2.5% Bi_2_O_3_/ZrO_2_ catalyst.

Fig. 4 illustrates the nitrogen adsorption–desorption isotherm of the Bi_2_O_3_/ZrO_2_ catalyst material. The N_2_ isotherm was associated to type IV, a typical H2-hysteresis loop, which describes characteristic mesoporous material lying within the *p*/*p*_o_ range of 0.59–0.97. The BET surface area of the 2.5% Bi_2_O_3_/ZrO_2_ catalyst material was shown to be 80.40 m^2^ g^−1^, pore volume 0.320 cm^3^ g^−1^ and pore size 106.4 Å. For the 1% Bi_2_O_3_/ZrO_2_ catalyst loading, the particles are small and have a high surface area, but had less active sites relative to the 2.5% Bi_2_O_3_/ZrO_2_. With the 5% Bi_2_O_3_/ZrO_2_ loading, the bismuth particles are visibly larger, and hence have a smaller surface area, when compared to the 2.5% loading and thus slightly lower yield. Hence, Bi_2_O_3_ on ZrO_2_ acts as a good promoter for the present transformation. These results suggest that bismuth on zirconia could act as a good promoter for the growth of additional crystalline faces, which cooperate to enhance the catalytic activity.

### 3.5 Pyridine IR analysis

**Fig. 5:**
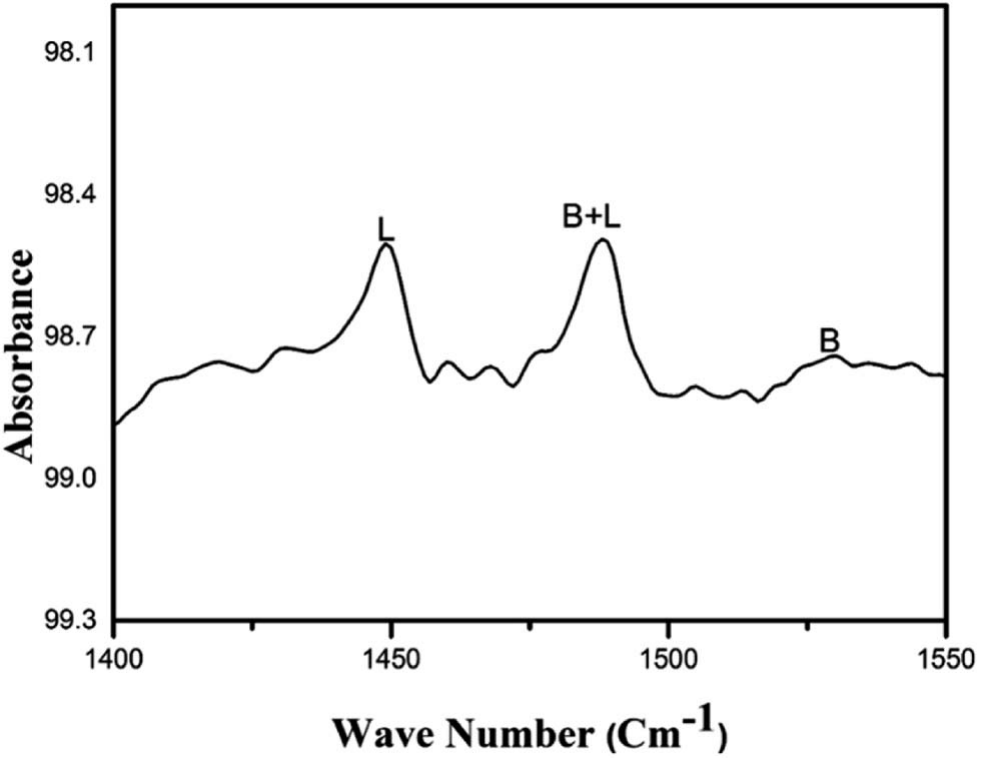
Pyridine IR spectrum of 2.5% Bi_2_O_3_/ZrO_2_ catalyst.

The *ex situ* pyridine^36^ adsorbed FT-IR spectrum in the range of 1600–1400 cm^−1^ for the prepared Bi_2_O_3_/ZrO_2_ is displayed in Fig. 5. The bands at 1449 cm^−1^, 1487 cm^−1^ and 1530 cm^−1^ were attributed to Lewis, Brønsted, and Lewis and Brønsted acidic sites respectively. Upon careful examination, the prepared catalyst material reveals strong Lewis acidic and weak Brønsted acidic sites.

### 3.6 Reaction optimization

The four-component reaction protocol of substituted aromatic aldehydes, malononitrile, hydrazine hydrate and diethyl acetylenedicarboxylate using a Bi_2_O_3_/ZrO_2_ catalyst is shown in Scheme 1. To optimise the reaction, decrease the reaction time and increase the product yield, the effects of variation of catalysts, solvents *etc.*, were examined on this model reaction. Initially, the reaction of 2-methoxy benzaldehyde, malanonitrile, hydrazine hydrate and diethyl acetylenedicarboxylate was performed under catalyst-free conditions. Only a trace of product was observed under RT reflux conditions or after 10 h of stirring (Table 1, entries 1 and 2). Different catalysts were employed in the presence of EtOH as the solvent at RT. The reaction was studied with commercially available acidic catalysts like acetic acid, FeCl_3_, and *p*-toluenesulfonic acid (PTSA). Low product yields were observed even after 9.5 h of stirring at RT (Table 1, entries 3–5). Next, trace amounts of yields were observed when the reaction was carried out with ionic liquids such as (Bmim)BF_4_ or l-proline (Table 1, entries 6 and 7) as catalysts. When the same reaction was performed in the presence of various basic organic and inorganic catalysts, such as TEA, pyridine, DABCO, NaOH and K_2_CO_3_ at RT, very low yields were observed (Table 1, entries 8–12). The reaction was conducted by using several pure metal oxide catalysts, such as SiO_2_, ZrO_2_ and Al_2_O_3_. Moderate yields were afforded at RT after 2.0–3.0 h reaction time (Table 1, entries 13–15). Among the selected heterogeneous catalysts, ZrO_2_ exhibited promising results with the highest yield (Table 1, entry 14). It is well known that mixed oxides are better catalysts compared to single oxides. Based on the results with ZrO_2_, to improve the yield and reaction times the reaction was attempted with various mixed metal oxides (2.5% CuO/ZrO_2_, MnO_2_/ZrO_2_, and Bi_2_O_3_/ZrO_2_), which all gave good to excellent yields (81–98%) at RT with EtOH as the solvent (Table 1, entries 16–18), and Bi proved to be superior. Hence, the effect of varied loading of Bi_2_O_3_ on ZrO_2_ was examined by using different wt% (1%, 2.5% and 5%) of Bi_2_O_3_ on ZrO_2_ supports; the results were impressive with excellent yields within short times (Table 1, entries 18–20). Using 1% Bi_2_O_3_ loaded on ZrO_2_ (Table 1, entry 19), the product yield was 90% in 45 min of stirring under the optimized conditions. A further increase of Bi loading (5%) led to a slightly decreased yield (96%) without any improvement in reaction time. While with 1% loading there were less active sites, the good activity with 2.5% loading may be because the dispersion of Bi_2_O_3_ on the surface of ZrO_2_ is uniform; with 5% loading, oligomerisation of Bi_2_O_3_ on the surface of ZrO_2_ may have happened, which decreases the activity of the sites. Thus, the catalytic activity was lower when compared with 2.5% loading. Based on this evaluation of the results, it is noticeable that 2.5% Bi_2_O_3_ loaded on zirconia catalyst has a higher surface area and subsequently the most reactive acidic sites owing to its nature and exhibited better catalytic activity compared to the other mixed catalysts. Furthermore, these catalysts have higher surface area, smaller particle sizes and more catalytic active sites than the related oxide homologues. Therefore, 2.5% Bi_2_O_3_/ZrO_2_ catalyst was preferred for all further reactions to attain excellent product yields.

**Table 1:** Optimal condition for the synthesis of 5a by 2.5% Bi_2_O_3_/ZrO_2_ catalyst^a^

Entry	Catalyst	Conditions	Time (h)	Yield^b^ (%)
1	—c	RT	10	11
2	—c	Reflux	10	12
3	AcOH	RT	9.5	36
4	FeCl_3_	RT	9.5	41
5	PTSA	RT	9.5	30
6	(Bmim)BF_4_	RT	8.0	6
7	l-Proline	RT	7.0	8
8	TEA	RT	6.0	17
9	Pyridine	RT	4.5	21
10	DABCO	RT	5.0	23
11	NaOH	RT	4.5	20
12	K_2_CO_3_	RT	4.0	25
13	SiO_2_	RT	3.0	49
14	ZrO_2_	RT	2.0	76
15	Al_2_O_3_	RT	2.5	58
16	2.5% CuO/ZrO_2_	RT	1.0	81
17	2.5% MnO_2_/ZrO_2_	RT	0.90	88
18	2.5% Bi_2_O_3_/ZrO_2_	RT	0.25	98
19	1% Bi_2_O_3_/ZrO_2_	RT	0.75	90
20	5% Bi_2_O_3_/ZrO_2_	RT	0.25	96

a All products were characterised by ^1^H-NMR, ^15^N NMR, ^13^C-NMR, HRMS and FT-IR spectral analyses.

b Isolated yields.

c — No catalyst used.

The model reaction with 2.5% Bi_2_O_3_/ZrO_2_ was conducted using varied non-polar and polar (protic and aprotic) solvents, such as *n*-hexane, toluene, THF, DMF, H_2_O, MeOH and EtOH, at RT (Table 2). No reaction was observed with non-polar solvents (*n*-hexane and toluene; Table 2, entries 1 and 2). However, polar aprotic solvents (THF and DMF) revealed a very low yield (Table 2, entries 3 and 4). Further, the reaction occurred efficiently with polar solvents (H_2_O, MeOH and EtOH) and with excellent yields in short reaction times except with H_2_O (Table 2, entries 5–7). When using H_2_O, as a polar green solvent, the reaction time increased and the yield was decreased. Based on these results, ethanol was chosen as the ideal solvent, which is also environmentally friendly and cost-effective.

**Table 2:** Optimization of solvent for the model reaction^a^

Entry	Solvent	Time (minutes)	Yield (%)
1	*n*-Hexane	120	—
2	Toluene	90	—
3	THF	60	8
4	DMF	60	12
5	H_2_O	60	45
6	MeOH	45	86
7	EtOH	15	98

a Reaction conditions: aromatic aldehydes (1 mmol), malononitrile (1 mmol), hydrazine hydrate (1 mmol), diethyl acetylenedicarboxylate (1 mmol) and solvent (5 mL) were stirred at room temperature.

Next, the model reaction was evaluated by employing different amounts of 2.5% Bi_2_O_3_/ZrO_2_ catalyst. The summarized outcomes (Table 3, entries 1–3) show that the increase in amount of catalyst from 10 mg to 30 mg leads to an increase in the product yield from 58% to 98% plus decreased reaction time. No significant change was observed in the yield of product with further increase in the amount of catalyst from 30 mg to 60 mg. Therefore, 30 mg of Bi_2_O_3_/ZrO_2_ catalyst was used for the further reactions.

**Table 3:** Optimization of various weight% for the model reaction by 2.5% Bi_2_O_3_/ZrO_2_ catalyst^a^

Entry	Catalyst (mg)	Time (min)	Yield (%)
1	10	90	58
2	20	45	80
3	30	15	98
4	40	15	98
5	50	15	98
6	60	20	98

a Reaction conditions: aromatic aldehydes (1 mmol), malononitrile (1 mmol), hydrazine hydrate (1 mmol), diethyl acetylenedicarboxylate (1 mmol), and solvent (5 mL) were stirred at room temperature.

For the optimised reaction conditions, to establish the wider scope of the protocol, the method was applied for the synthesis of different pyranopyrazoles using various substituted aromatic aldehydes (Table S4†) and the results are summarized in Table 4. The 2.5% Bi_2_O_3_/ZrO_2_ catalyst material catalysed the facile one-pot synthesis of pyranopyrazole derivatives with excellent yields in short reaction times (<20 min). Remarkably, the aldehydes with both electron donating and electron withdrawing (*ortho*, *meta* and *para*) substituents worked efficiently under the reaction conditions, producing the corresponding target products (5a–k).

**Table 4:** Synthesis of novel functionalized pyridine derivatives by 2.5% Bi_2_O_3_/ZrO_2_ catalyst^a^

Entry	R	Product	Yield (%)	Mp °C
1	2-OMe	5a	97	205–207
2	4-OMe	5b	98	208–210
3	2,3-OMe	5c	94	230–232
4	3,4-OMe	5d	91	243–245
5	2,5-OMe	5e	93	257–259
6	2,4,6-OMe	5f	96	240–242
7	3-OH	5g	92	221–223
8	3,4-OH	5h	98	204–206
9	2-NO_2_	5i	95	235–237
10	4-Br	5j	94	225–227
11	4-Et	5k	96	210–212

a Reaction conditions: aromatic aldehydes (1 mmol), malononitrile (1 mmol), hydrazine hydrate (1 mmol), diethyl acetylenedicarboxylate (1 mmol), and solvent (5 mL) were stirred at room temperature.

## 4. Reusability of catalyst

The reusability and recyclability of a solid catalyst material is an important parameter as per green chemistry principles. Several recycling experiments were conducted to examine the stability and sustainability of the catalyst material. After completion of every run, filtration was employed to separate the catalyst from the crude product.

Then the catalyst was washed with ethanol and dried at 120 °C for 3 h for up to seven runs. Marginal loss of less than 5% of the catalyst was observed in the recovery procedure. Then it was washed with ethanol and dried at 120 °C for 3 h. The loss was supplemented to 30 mg by adding the minute amount required. Activity was retained with no loss in the first six runs, then the material's catalytic activity weakened by 4% in the 7th cycle. No loss of catalytic activity could be observed up to the 6th run owing to the minor losses in the recovery process and non-leaching of the active material.

## 5. Mechanism

**Scheme 2:**
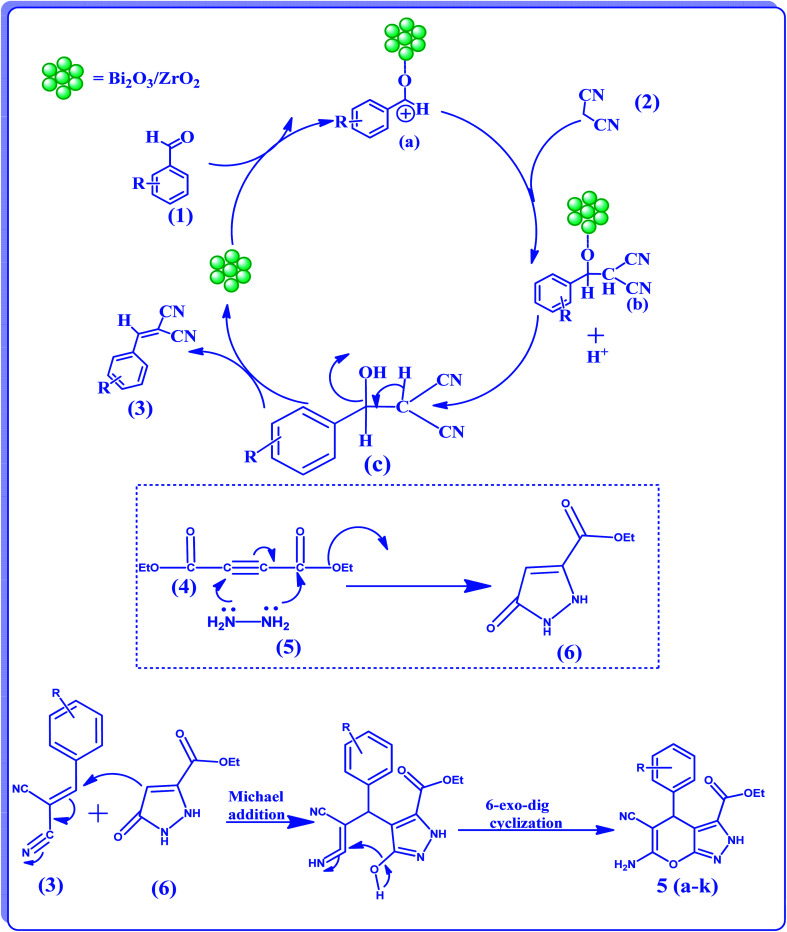
Plausible reaction mechanism for the formation of 2,4-dihydropyrano[2,3-*c*] pyrazole-3-carboxylate derivatives.

In agreement with experimental results, a plausible mechanism is suggested in Scheme 2. The presence of Lewis acidity on the catalyst surface would facilitate the reaction. It may be assumed that in the first step Knoevenagel condensation^37^ is achieved by the coordination of Lewis acidic sites with the oxygen of the carbonyl group, forming a carbocation intermediate (a). In the next step, the active methylene group reacts with the carbocation intermediate giving (b); next it will dissociate from the catalyst surface taking a proton from the protic solvent (EtOH) and giving (c). It will further undergo dehydration giving (3). In the next step, ethyl 5-oxo-2,5-dihydro-1H-pyrazole-3-carboxylate (6) is possibly formed by the reaction between hydrazine hydrate (5) with diethyl acetylenedicarboxylate (4). Finally, a Michael addition between (3) and (6) occurs, yielding the desired product selectively through 6-*exo*-dig cyclization. The catalytic efficiency of the Bi_2_O_3_/ZrO_2_ on the title reaction in comparison with other reported catalysts is summarized in the Table 5.

**Table 5:** Comparison of various catalysts for the synthesis of pyrano[2,3-*c*]pyrazole derivatives

Catalyst	Solvent	Reaction condition	Time	Yield (%) [ref.]
Silica-supported tetramethylguanidine	Neat	RT	15–60 min	79–98 (ref. 22)
BS-2G-Ti	H_2_O	Heating	60–100 min	45–96 (ref. 23)
Ba(OH)_2_	H_2_O	Reflux	60–120 min	81–93 (ref. 24)
γ-Alumina	H_2_O	Reflux	35–90 min	61–90 (ref. 25)
Amberlyst A21	EtOH	RT	10–65 min	73–98 (ref. 26)
AcOH	AcOH	Reflux	72–90 min	71–92 (ref. 27)
Visible light assisted	Neat	RT	15–25 min	56–90 (ref. 28)
2.5% Bi_2_O_3_–ZrO_2_	EtOH	RT	≈20 min	91–98 [this work]

## 6. Conclusion

In summary, we designed a highly efficient and cost-effective method for the synthesis of pyranopyrazole derivatives *via* a one-pot, four-component reaction in ethanol as a green solvent, using environmentally benign Bi_2_O_3_/ZrO_2_ as a selective catalyst. Of the 11 derivatives synthesised, eight are new molecules. The operational simplicity, short reaction times, high yields, eco-friendly solvent, and mild reaction conditions make this method attractive. Additionally, the catalyst can be easily recovered and recycled for at least six runs without loss of efficiency. Moreover, expansion of the reaction scope and synthetic and medicinal applications of this methodology are in progress in our laboratory.

## Conflicts of interest

There are no conflicts to declare.

## Supplementary Material

RA-008-C8RA01994K-s001
