# Supplementary material for: An efficient and green approach for the synthesis of 2,4-dihydropyrano[2,3-c]pyrazole-3-carboxylates using Bi2O3/ZrO2 as a reusable catalyst
Source: RSC Adv. 2018 May 3;8(29):16336–43. doi: 10.1039/c8ra01994k (PMC9080279; doi:10.1039/c8ra01994k)
Supplement: RA-008-C8RA01994K-s001 [file RA-008-C8RA01994K-s001.pdf]

Electronic Supplementary Material (ESI) for RSC Advances.
This journal is © The Royal Society of Chemistry 2018

An efficient, green approach for the synthesis of 2,4-dihydropyrano[2,3-c] pyrazole-3-carboxylates using Bi_2O_3/ZrO_2 as a reusable catalyst

Sandeep V.H.S. Bhaskaruni, Suresh Maddila, Werner E. van Zyl and Sreekantha B. Jonnalagadda*

*School of Chemistry & Physics, University of KwaZulu-Natal, Westville Campus, Chiltern Hills, Durban-4000, South Africa

*Corresponding Author: Prof. Sreekantha B. Jonnalagadda
School of Chemistry & Physics,
University of KwaZulu-Natal,
Durban 4000, South Africa.
Tel.: +27 31 2607325,
Fax: +27 31 2603091
E-mail address: jonnalagaddas@ukzn.ac.za

Contents	Pages
Catalyst instrumentation details (SI – I)	2
Materials, methods and instruments (SI – I)	2
Spectral characterization data (SI – II)	3-5
All the synthesized spectral information (SI – III)	6-37

Catalyst instrumentation details

Employing a Bruker D8 Advance instrument (Cu K radiation source with a wave length of 1.5406 Å), the X-ray diffraction data related the structural phases of the catalyst were acquired. Using a Jeol JEM-1010 electron microscope and JEOL JSM-6100 microscope, the TEM and SEM analysis data was recorded. iTEM software was used analyze the TEM data and images. Employing the X-ray analyzer (energy-dispersive), EDX-analysis on the SEM images was conducted.

Experimental Section:

All chemicals and reagents required for the reaction were of analytical grade and were used without any further purification. Bruker AMX 400 MHz NMR spectrometer was used to record the ^{1}H NMR, ^{13}C NMR and ^{15}N NMR spectral values. High-resolution mass data were obtained using a Bruker micro TOF-Q II ESI instrument operating at ambient temperature. The DMSO−d_6 solution was utilized for this while TMS served as the internal standard. TMS was further used as an internal standard for reporting the all chemical shifts in δ (ppm). Purity of all the reaction products was confirmed by TLC using aluminum plates coated with silica gel (Merck Kieselgel 60 F254).

Ethyl 6-amino-5-cyano-4-(2-methoxyphenyl)-2,4-dihydropyrano[2,3-c]pyrazole-3-carboxylate (5a):

^{1}H NMR (400 MHz, DMSO-d_6) δ = 1.01 (t, *J* = 7.12 Hz, 3H, CH_3), 3.69 (s, 3H, OCH_3), 4.01-4.06 (m, 2H, CH_2), 5.05 (s, 1H, CH), 6.82 (d, *J* = 8.56 Hz, 2H, ArH), 6.85 (s,2H, NH_2), 6.89 (dd, *J* = 1.56 Hz, *J* = 1.56 Hz, 1H, ArH), 6.94 (d, *J* = 8.04, 1H, ArH), 7.15-7.22 (m, 1H, ArH), 13.57 (s, 1H, NH); ^{13}C NMR (100 MHz, DMSO-d_6): 13.57, 31.62, 55.43, 56.00, 60.62, 103.58, 111.33, 120.21, 127.87, 128.59, 132.41, 136.46, 156.25, 156.60, 158.25, 160.56; ^{15}N NMR (40.55 MHz, DMSO-d_6) δ = 6.85 (s, 2H, NH_2), 13.57 (s, 1H, NH). HRMS of [$C_{17}H_{16}N_4O_4$–H^+] (m/z): 339.1082; Calcd.: 339.0992.

Ethyl 6-amino-5-cyano-4-(4-methoxyphenyl)-2,4-dihydropyrano[2,3-c]pyrazole-3-carboxylate (5b):

^{1}H NMR (400 MHz, DMSO-d_6) δ = 1.08 (t, *J* = 7.08Hz, 3H, CH_3), 3.70 (s, 3H, OCH_3), 4.07-4.12 (m, 2H, CH_2), 4.69 (s, 1H, CH), 6.83 (d, *J* = 8.56 Hz, 2H, ArH), 6.97 (s,2H, NH_2), 7.00 (d, *J* = 8.6 Hz, 2H, ArH), 13.69 (s, 1H, NH); ^{13}C NMR (100 MHz, DMSO-d_6): 13.77, 36.16, 54.95, 58.16, 60.79, 113.53, 115.16, 120.31, 124.08, 128.31, 133.32, 137.07, 157.87, 158.12, 159.87. ^{15}N NMR (40.55 MHz, DMSO-d_6) δ = 6.97 (s, 2H, NH_2), 13.69 (s, 1H, NH). HRMS of [$C_{17}H_{16}N_4O_4$–H^+] (m/z): 339.1082; Calcd.: 339.1093.

Ethyl 6-amino-5-cyano-4-(2,3-dimethoxyphenyl)-2,4-dihydropyrano[2,3-c]pyrazole-3-carboxylate (5c):

^{1}H NMR (400 MHz, DMSO-d_6) δ = 1.06 (t, *J* = 7.04 Hz, 3H, CH_3),3.54 (s, 3H, OCH_3), 3.76 (s, 3H, OCH_3), 4.07-4.09 (m, 2H, CH_2), 4.91 (s, 1H, CH), 6.60 (d, *J* = 7.13 Hz, 2H, ArH), 6.89 (d, *J* = 8.28 Hz, 1H, ArH), 6.91 (s, 2H, NH_2), 6.95 (d, *J* = 7.92 Hz, 1H, ArH), 13.55 (s, 1H, NH); ^{13}C NMR (100 MHz, DMSO-d_6): 13.72, 32.79, 55.48, 56.90, 59.69, 60.67, 103.87, 111.46, 120.56, 121.37, 123.18, 128.68, 137.21, 146.42, 152.26, 155.91, 158.19, 160.48; ^{15}N NMR (40.55 MHz, DMSO-d_6) δ = 6.91 (s, 2H, NH_2), 13.55 (s, 1H, NH). HRMS of [$C_{18}H_{18}N_4O_5$+Na^+] (m/z): 393.1188; Calcd.: 393.1175.

Ethyl 6-amino-5-cyano-4-(3,4-dimethoxyphenyl)-2,4-dihydropyrano[2,3-c]pyrazole-3-carboxylate (5d):

^{1}H NMR (400 MHz, DMSO-d_6) δ = 1.08 (t, *J* = 7.12 Hz, 3H, CH_3), 3.68 (d, *J* = 5.72 6H, OCH_3), 4.08-4.13 (m, 2H, CH_2), 4.70 (s, 1H, CH), 6.56 (dd, *J* = 1.84 Hz, *J* = 1.84, 1H, ArH), 6.71 (d, *J* = 1.84 Hz, 1H, ArH), 6.55 (d, *J* = 8.32 Hz, 1H, ArH), 6.96 (s, 2H, NH_2), 13.55 (s, 1H, NH); ^{13}C NMR (100 MHz, DMSO-d_6): 13.76, 36.46, 55.44, 58.04, 60.79, 103.84, 111.73, 119.22, 127.24, 128.93, 137.55, 148.25, 154.42, 155.48, 158.15, 159.93, 160.59; ^{15}N NMR (40.55 MHz, DMSO-d_6) δ = 6.96 (s, 2H, NH_2), 13.55 (s, 1H, NH). HRMS of [$C_{18}H_{19}N_4O_5$+H^+] (m/z): 371.0389; Calcd.: 371.0395.

Ethyl 6-amino-5-cyano-4-(2,5-dimethoxyphenyl)-2,4-dihydropyrano[2,3-c]pyrazole-3-carboxylate (5e):

^{1}H NMR (400 MHz, DMSO-d$_6$) δ = 1.04 (t, *J* = 7.12 Hz, 3H, CH$_3$), 3.61 (d, *J* = 6.61 Hz, 6H, OCH3), 4.03-4.09 (m, 2H, CH$_2$), 4.97 (s, 1H, CH), 6.46 (d, *J* = 2.66 Hz, 1H, ArH), 6.74 (dd, *J* = 3 Hz, *J* = 3.04Hz, 1H, ArH), 6.85 (s, 1H, ArH), 6.87 (s, 2H, NH$_2$), 13.58 (s, 1H, NH); ^{13}C NMR (100 MHz, DMSO-d$_6$): 13.58, 32.12, 55.18, 56.05, 56.57, 60.69, 103.39, 111.52, 112.60, 115.48, 120.33, 128.61, 133.54, 150.99, 152.82, 156.18, 158.25, 160.62; ^{15}N NMR (40.55 MHz, DMSO-d$_6$) δ = 6.87 (s, 2H, NH$_2$), 13.58 (s, 1H, NH).

Ethyl 6-amino-5-cyano-4-(2,4,6-trimethoxyphenyl)-2,4-dihydropyrano[2,3-c]pyrazole-3-carboxylate (5f):

^{1}H NMR (400 MHz, DMSO-d$_6$) δ = 1.06 (t, *J* = 7.09 Hz, 3H, CH$_3$), 3.73 (s, 3H, OCH$_3$), 3.89 (d, *J* = 7.96 Hz, 6H, OCH$_3$), 4.02-4.11 (m, 2H, CH$_2$), 5.27 (s, 1H, CH), 6.33 (s, 2H, ArH), 6.88 (s,2H, NH$_2$), 13.25 (s, 1H, NH); ^{13}C NMR (100 MHz, DMSO-d$_6$): 13.78, 25.41, 55.04, 55.79, 56.11, 60.38, 103.69, 104.51, 112.39, 113.84, 116.38, 120.70, 156.67, 158.59, 159.55, 167.37. ;^{15}N NMR (40.55 MHz, DMSO-d$_6$) δ = 6.88 (s, 2H, NH$_2$), 13.25 (s, 1H, NH). HRMS of [$C_{19}H_{20}N_4O_6$+Na^+] (m/z): 423.1297; Calcd.: 423.1281.

Ethyl 6-amino-5-cyano-4-(3-hydroxyphenyl)-2,4-dihydropyrano[2,3-c]pyrazole-3-carboxylate (5g):

^{1}H NMR (400 MHz, DMSO-d$_6$) δ = 1.07 (t, *J* = 7.08 Hz, 3H, CH$_3$), 4.08-4.13 (m, 2H, CH$_2$), 4.63 (s, 1H, CH), 6.40 (s, 1H, ArH), 6.51-6.58 (m, 2H, ArH), 6.99 (s, 2H, NH$_2$), 7.05 (t, *J* = 7.79 Hz,1H, ArH), 9.27 (s, 1H, OH), 13.71 (s, 1H, NH); ^{13}C NMR (100 MHz, DMSO-d$_6$): 13.74, 36.85, 55.98, 60.80, 103.74, 113.59, 114.06, 117.99, 120.26, 128.95, 129.07, 146.30, 155.56, 157.16, 158.16, 160.02;^{15}N NMR (40.55 MHz, DMSO-d$_6$) δ = 6.99 (s, 2H, NH$_2$), 13.71 (s, 1H, NH)

Ethyl 6-amino-5-cyano-4-(3,4-dihydroxyphenyl)-2,4-dihydropyrano[2,3-c]pyrazole-3 carboxylate (5h):

^{1}H NMR (400 MHz, DMSO-d$_6$) δ = 1.12 (t, *J* = 7.12 Hz, 3H, CH$_3$), 4.10-4.15 (m, 2H, CH$_2$), 4.54 (s, 1H, CH), 6.37-6.42 (m, 2H, ArH), 6.60 (d, *J* = 8.04 Hz, 1H, ArH), 6.93 (s, 2H, NH$_2$), 8.67 (s, 1H, OH), 8.79 (s, 1H, OH), 13.65 (s, 1H, NH); ^{13}C NMR (100 MHz, DMSO-d$_6$): 13.82, 36.34, 58.42, 60.80, 104.41, 114.50, 115.04, 118.12, 120.41, 128.81, 136.05, 143.91, 144.55, 155.47, 158.23, 159.83; ^{15}N NMR (40.55 MHz, DMSO-d$_6$) δ = 6.93 (s, 2H, NH$_2$), 13.65 (s, 1H, NH). HRMS of [$C_{16}H_{14}N_4O_5$—H^+] (m/z): 341.0886; Calcd.: 341.0886.

Ethyl 6-amino-5-cyano-4-(2-nitrophenyl)-2,4-dihydropyrano[2,3-c]pyrazole-3-carboxylate (5i):

^{1}H NMR (400 MHz, DMSO-d$_6$) δ = 0.92 (t, *J* = 7.08 Hz, 3H, CH$_3$), 3.96-4.05 (m, 2H, CH$_2$), 5.57 (s, 1H, CH), 7.14 (s, 2H, NH$_2$), 7.22 (dd, *J* = 1.08, *J* = 1.08 Hz, 1H, ArH), 7.44-7.48 (m,

1H, ArH), 7.61-7.65 (m, 1H, ArH), 7.92 (dd, *J* = 1.12 Hz, *J* = 1.12 Hz, ArH), 13.79 (s, 1H, NH); ^{13}C NMR (100 MHz, DMSO-d_6): 13.63, 31.49, 56.12, 60.85, 102.33, 119.60, 128.02, 128.99, 131.07, 133.64, 138.89, 148.20, 155.58, 157.73, 160.55; ^{15}N NMR (40.55 MHz, DMSO-d_6) δ = 7.14 (s, 2H, NH_2), 13.79 (s, 1H, NH). HRMS of [$C_{16}H_{13}N_5O_5$—H^+] (m/z): 354.0850; Calcd.: 354.0838.

Ethyl 6-amino-4-(4-bromophenyl)-5-cyano-2,4-dihydropyrano[2,3-c]pyrazole-3-carboxylate (5j):

^{1}H NMR (400 MHz, DMSO-d_6) δ = 1.05 (t, *J* = 3.56 Hz, 3H, CH_3), 4.06-4.12 (m, 2H, CH_2), 4.76 (s, 1H, CH), 7.05 (s, 2H, ArH), 7.07 (s, 2H, NH_2), 7.47 (d, *J* = 8.32, 2H, ArH), 13.77 (s, 1H, NH); ^{13}C NMR (100 MHz, DMSO-d_6): 13.76, 36.37, 57.27, 60.85, 102.99, 119.59, 120.09, 129.06, 129.62, 131.11, 144.26, 155.45, 157.97, 160.00; ^{15}N NMR (40.55 MHz, DMSO-d_6) δ = 7.07 (s, 2H, NH_2), 13.77 (s, 1H, NH). HRMS of [$C_{16}H_{13}BrN_4O_3$—H^+] (m/z): 387.0106; Calcd.: 387.0093.

Ethyl 6-amino-5-cyano-4-(4-ethylphenyl)-2,4-dihydropyrano[2,3-c]pyrazole-3-carboxylate (5k):

^{1}H NMR (400 MHz, DMSO-d_6) δ = 1.06 (d, *J* = 6.44 Hz, 3H, CH_3), 1.15 (d, *J* = 6.44 Hz, 3H, CH_3), 2.54 (d, *J* = 7.04 Hz, 2H, CH_2), 4.09 (d, *J* = 5.56 Hz, 2H, CH_2), 4.70 (s, 1H, CH), 6.99 (s, 2H, NH_2), 7.11 (d, *J* = 6.60 Hz,4H, ArH), 13.70 (s, 1H, NH); ^{13}C NMR (100 MHz, DMSO-d_6): 13.71, 15.49, 27.70, 36.55, 57.95, 60.76, 103.83, 120.31, 127.17, 127.55, 128.91, 141.93, 142.23, 155.54, 158.12, 159.97; ^{15}N NMR (40.55 MHz, DMSO-d_6) δ = 6.99 (s, 2H, NH_2), 13.70 (s, 1H, NH). HRMS of [$C_{18}H_{18}N_4O_3$—H^+] (m/z): 337.1310; Calcd.: 337.1301.

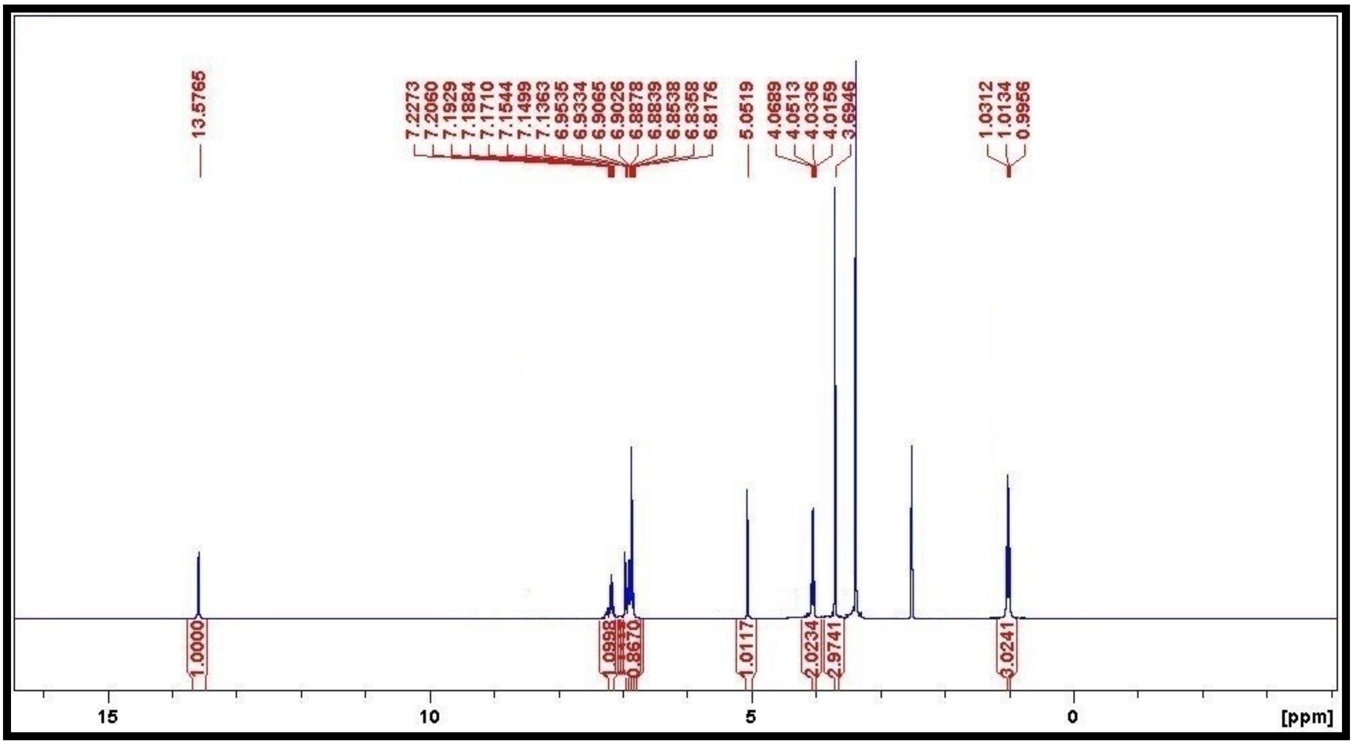

^{1}H NMR spectra of compound **5a**

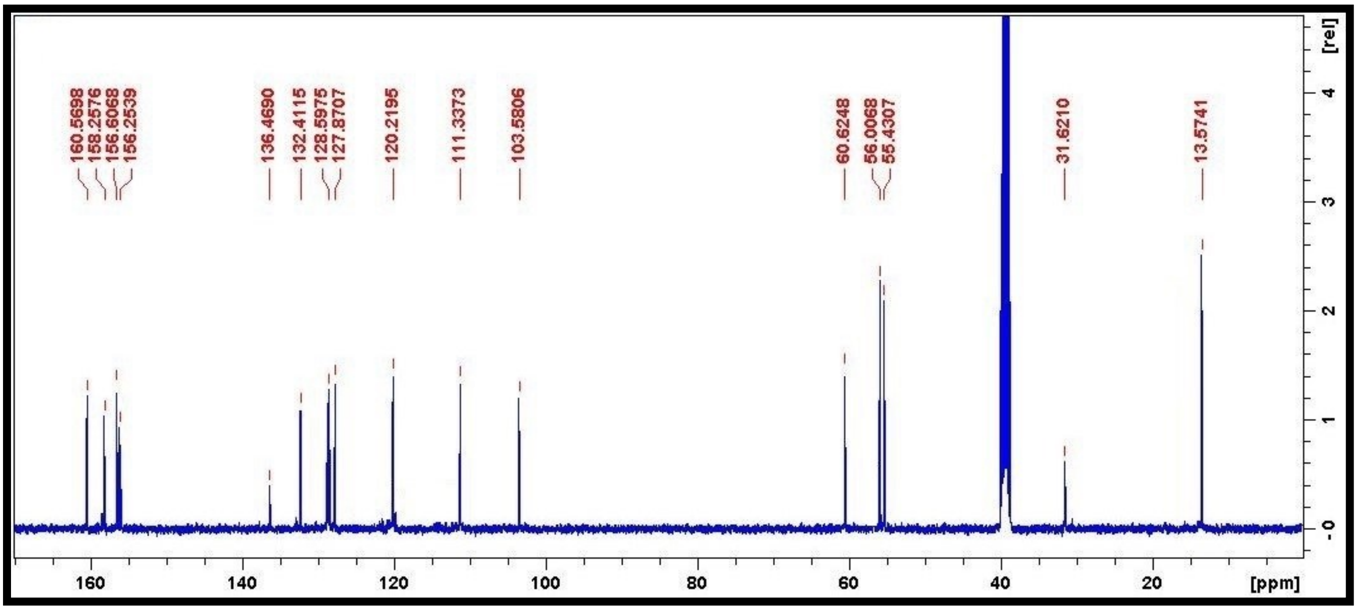

^{13}C NMR spectra of compound **5a**

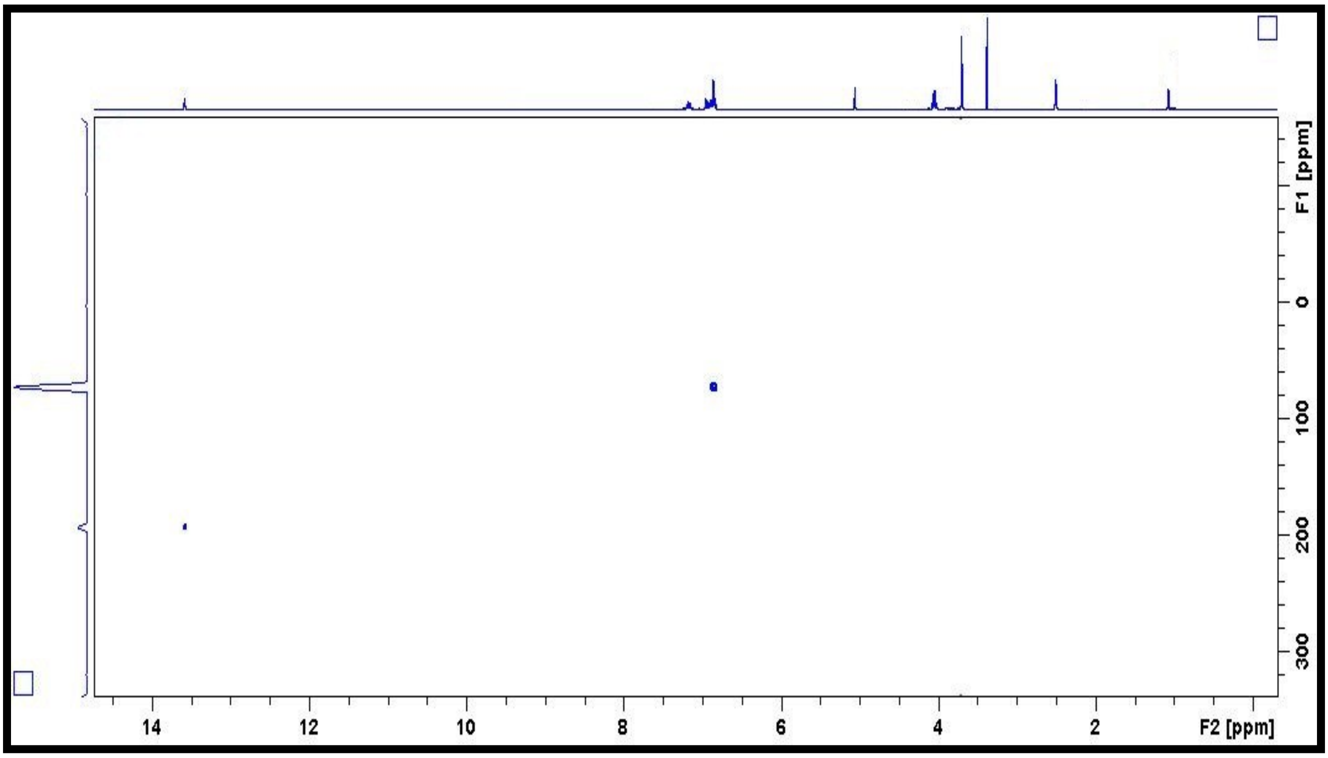

^{15}N NMR spectra of compound **5a**

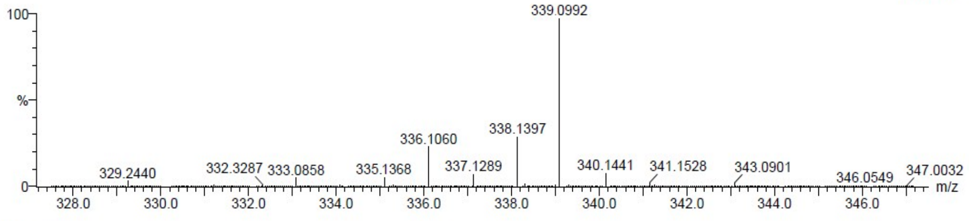

Elemental Composition Report **Page 1**

Single Mass Analysis
Tolerance = 5.0 PPM / DBE: min = -1.5, max = 100.0
Element prediction: Off
Number of isotope peaks used for i-FIT = 2

Monoisotopic Mass, Even Electron Ions
27 formula(e) evaluated with 1 results within limits (up to 20 best isotopic matches for each mass)
Elements Used:
C: 15-20 H: 15-20 N: 0-5 O: 0-5

Compound 2 2 (0.034) Cm (1:61)
TOF MS ES-

Minimum:				-1.5			
Maximum:		5.0	5.0	100.0			
Mass	Calc. Mass	mDa	PPM	DBE	i-FIT	i-FIT (Norm)	Formula
339.0992	339.1008	-1.6	-4.8	10.5	700.0	0.0	C17 H15 N4 O4

HRMS spectra of compound **5a**

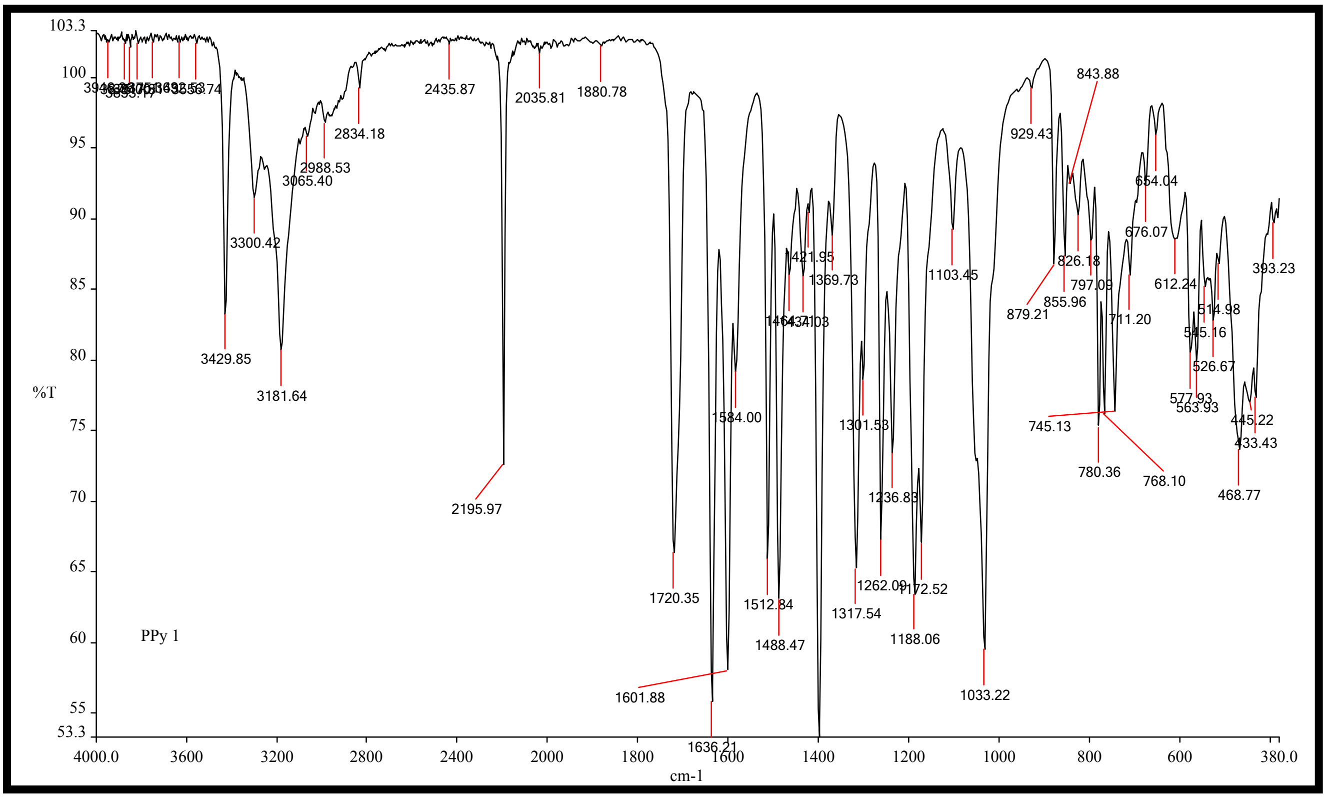

FT-IR spectra of compound **5a**

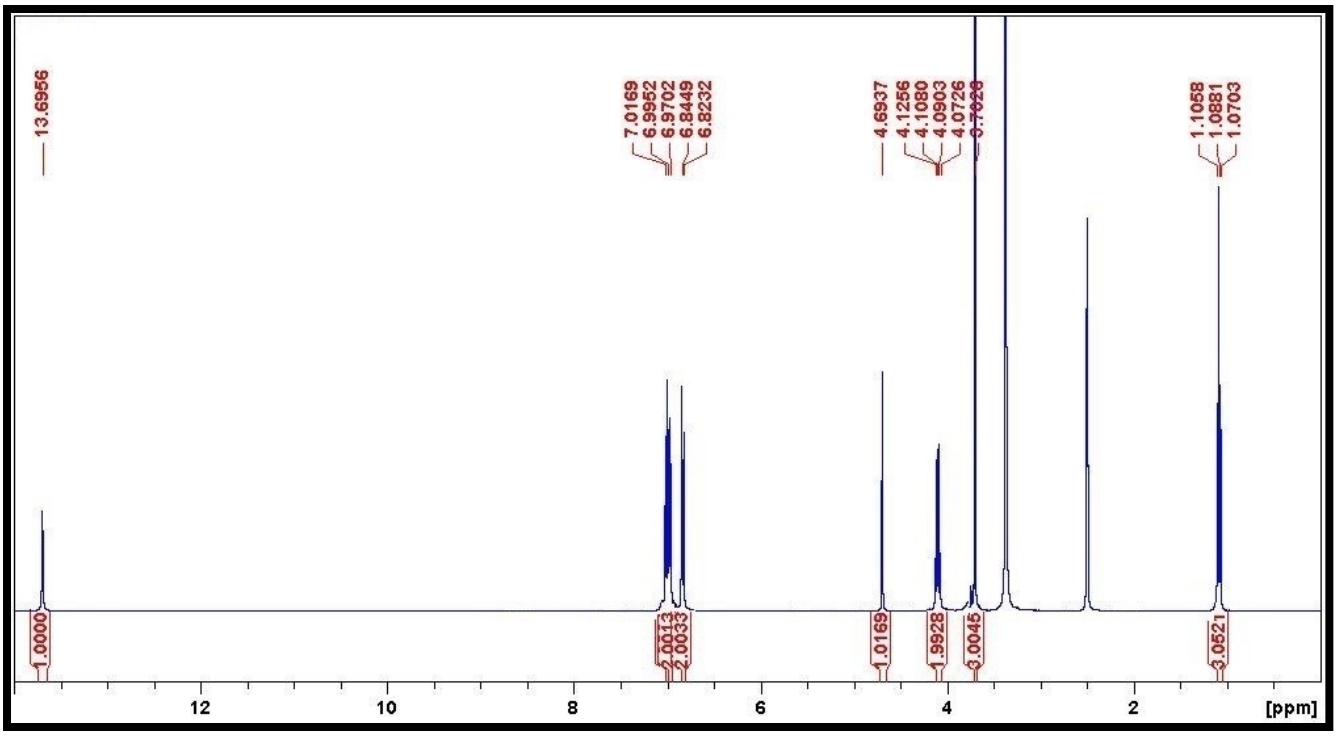

^{1}H NMR spectra of compound **5b**

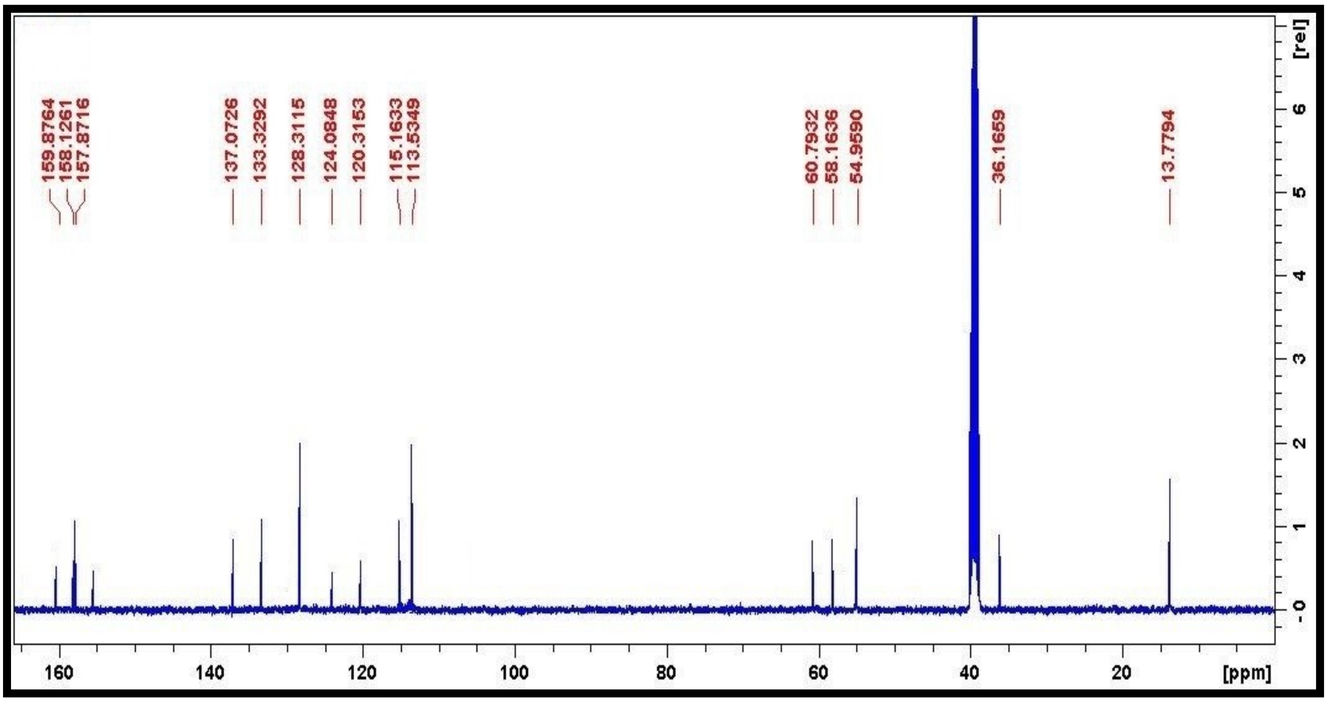

^{13}C NMR spectra of compound **5b**

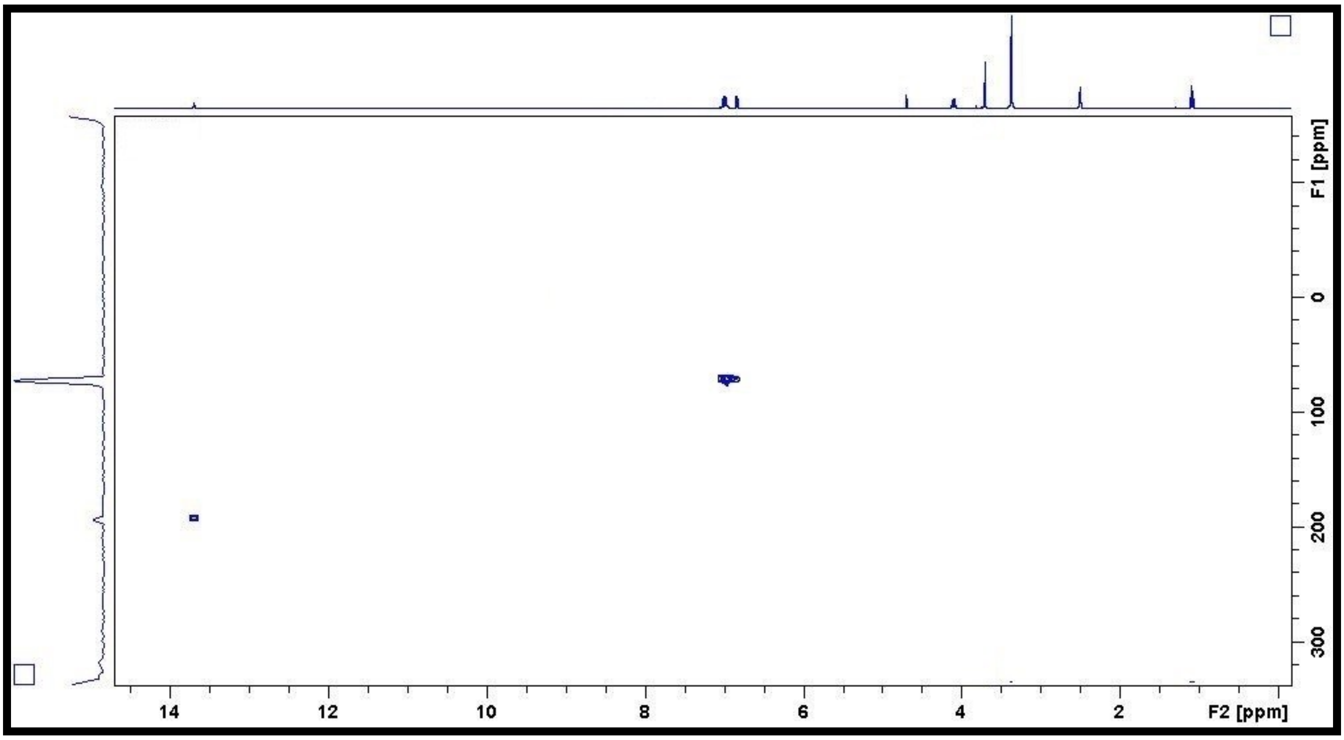

^{15}N NMR spectra of compound **5b**

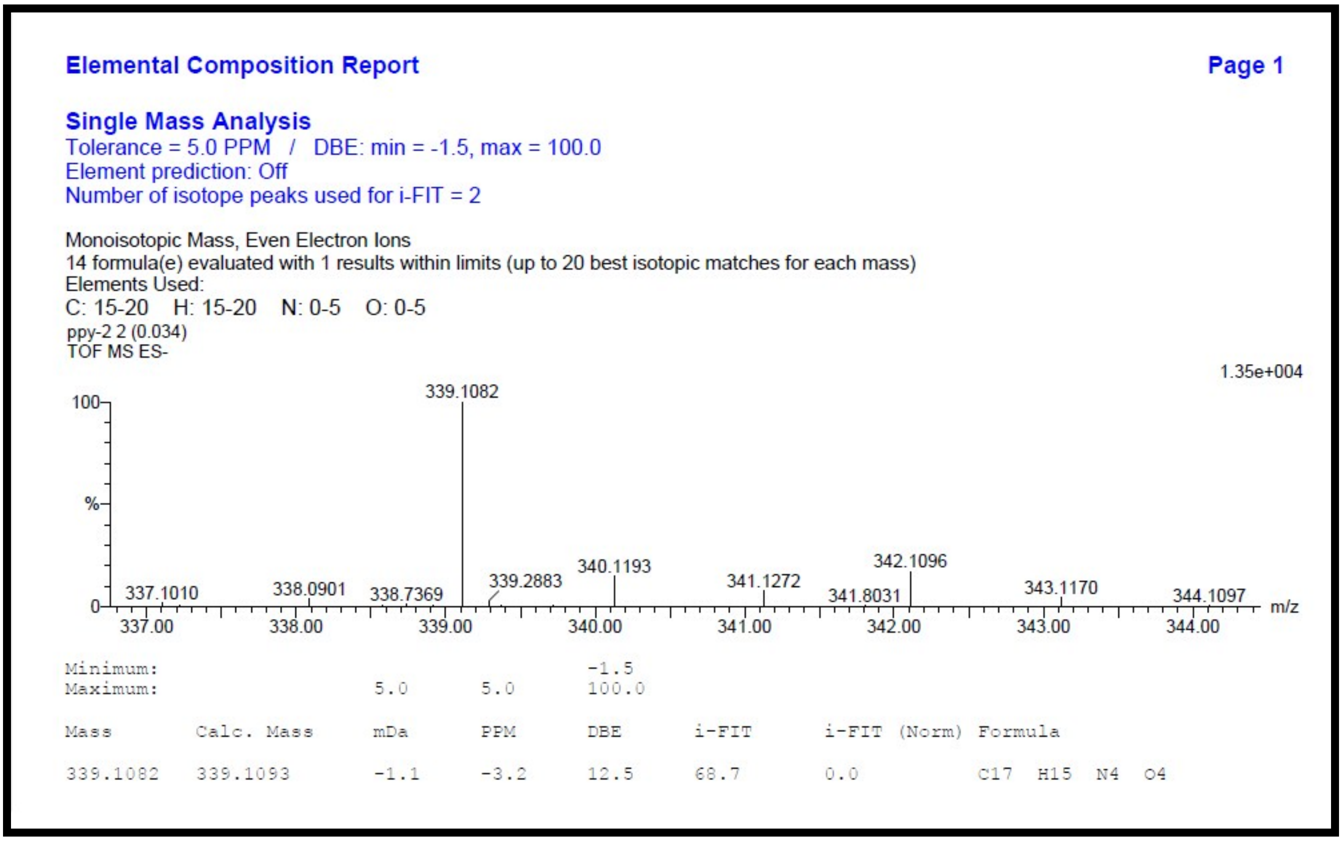

HRMS spectra of compound **5b**

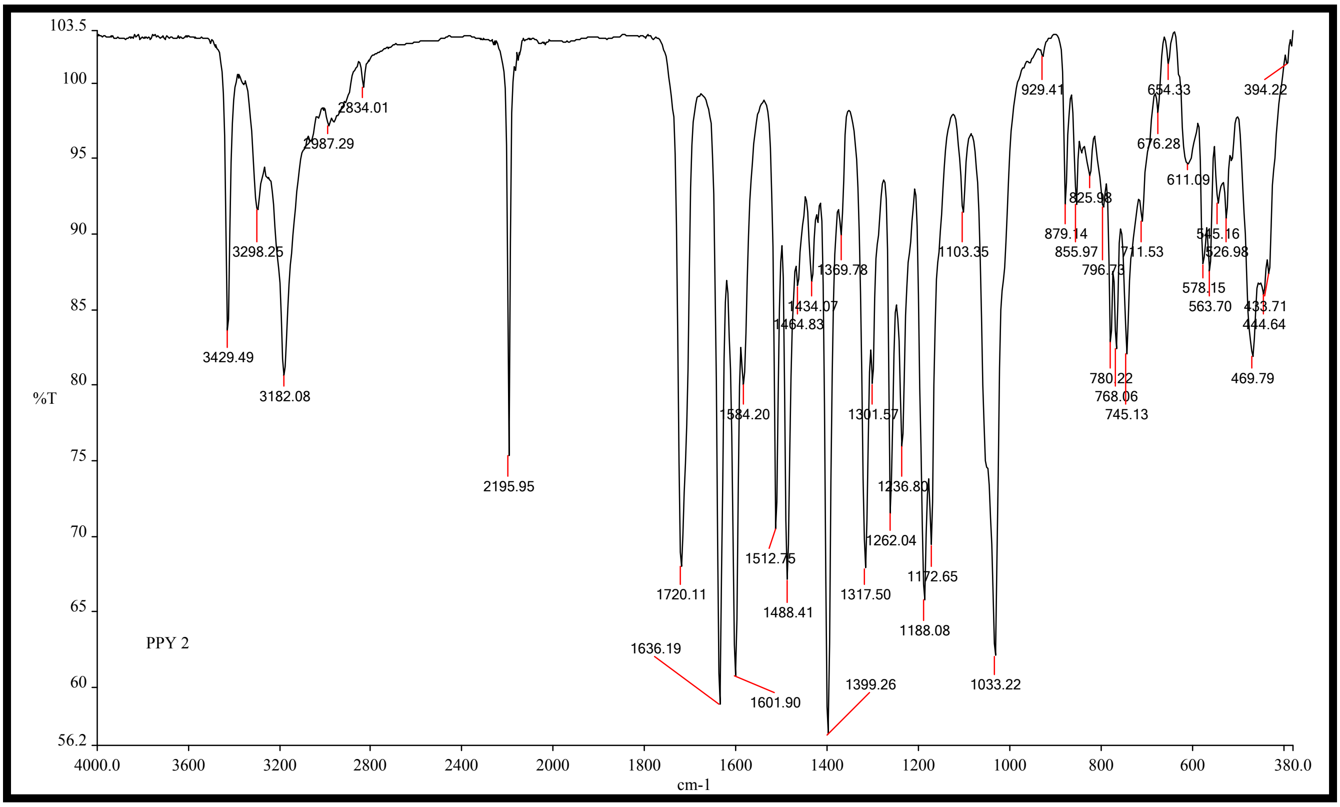

FT-IR spectra of compound **5b**

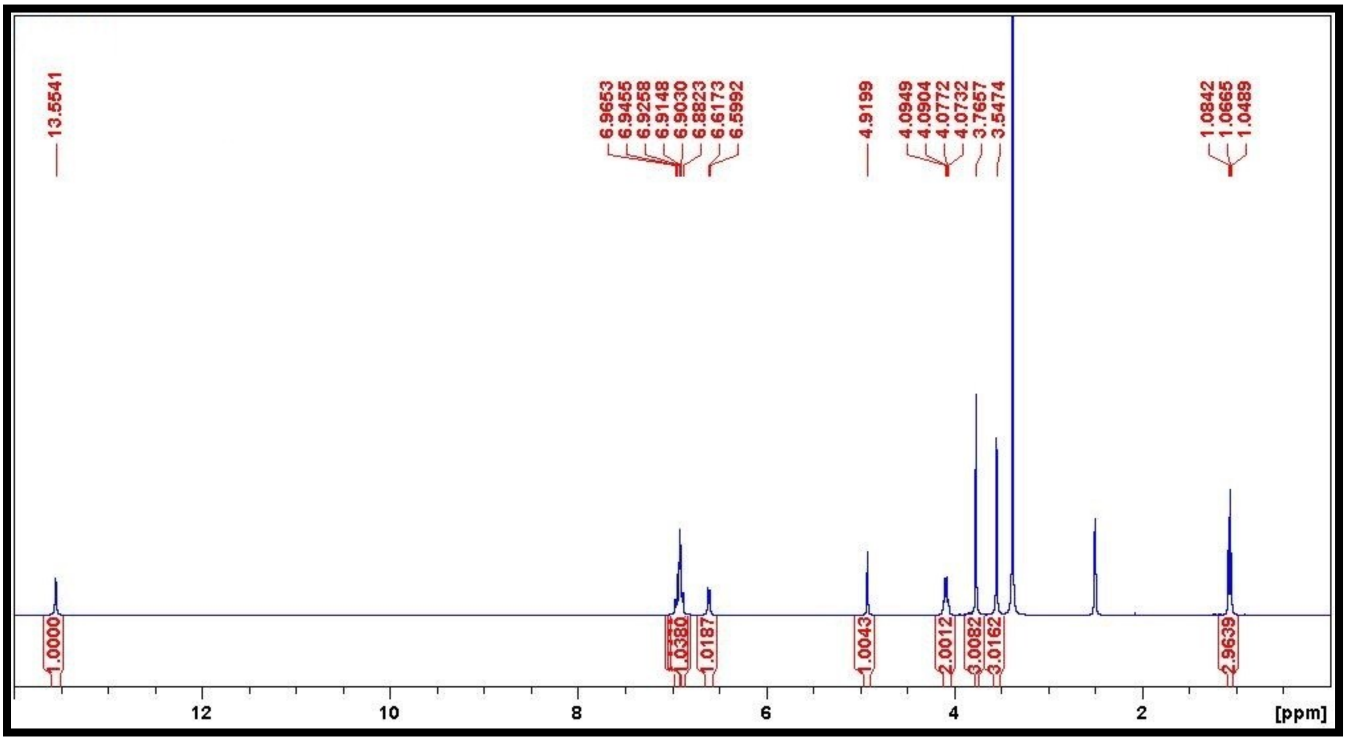

^{1}H NMR spectra of compound **5c**

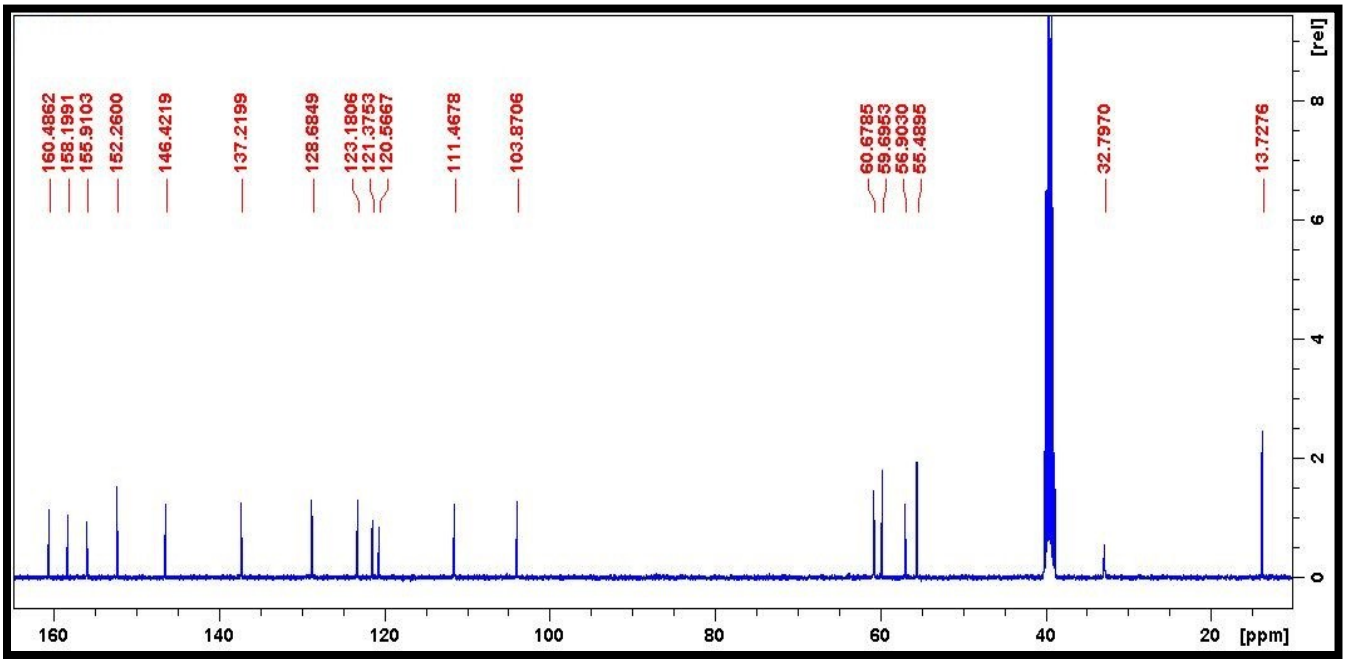

^{13}C NMR spectra of compound **5c**

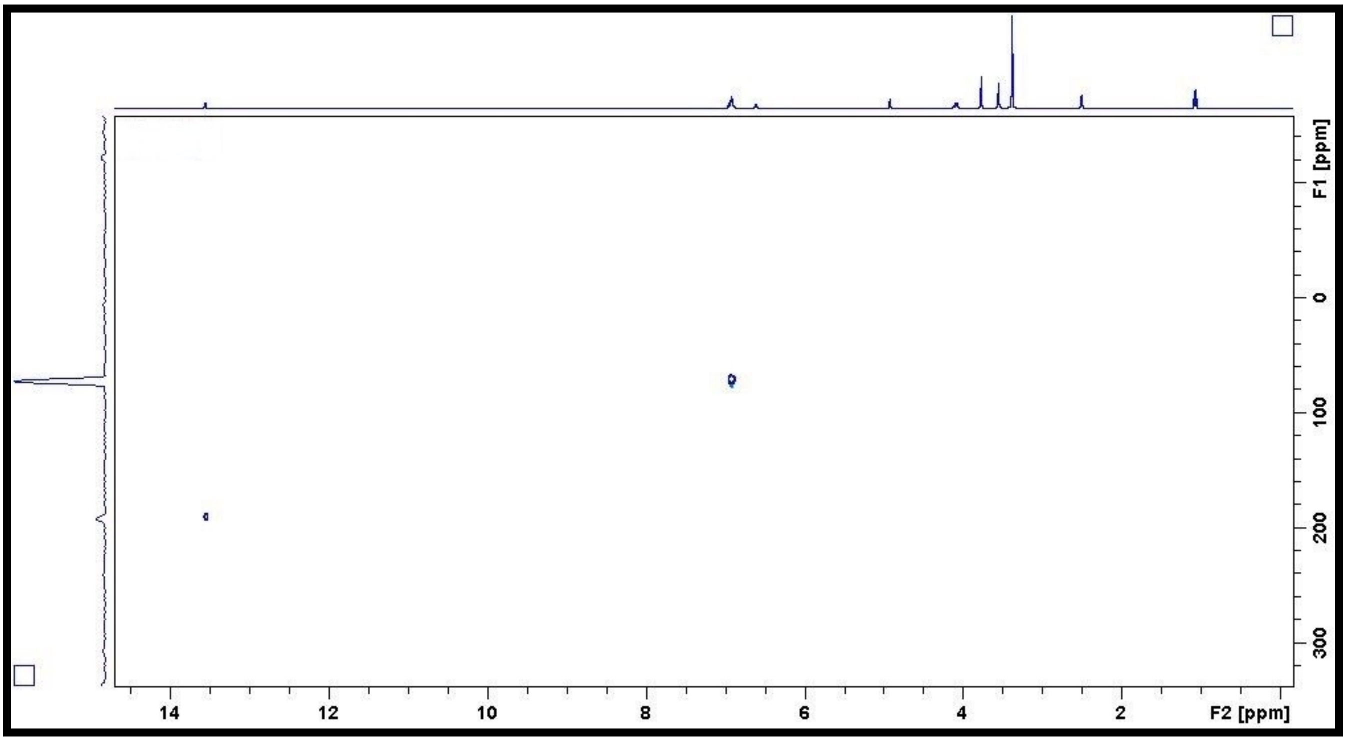

^{15}N NMR spectra of compound **5c**

Elemental Composition Report **Page 1**

Single Mass Analysis
Tolerance = 5.0 PPM / DBE: min = -1.5, max = 100.0
Element prediction: Off
Number of isotope peaks used for i-FIT = 2

Monoisotopic Mass, Even Electron Ions
19 formula(e) evaluated with 1 results within limits (up to 20 best isotopic matches for each mass)
Elements Used:
C: 15-20 H: 15-20 N: 0-5 O: 0-5 Na: 1-1
ppy-3 57 (1.889) Cm (1:61)
TOF MS ES+

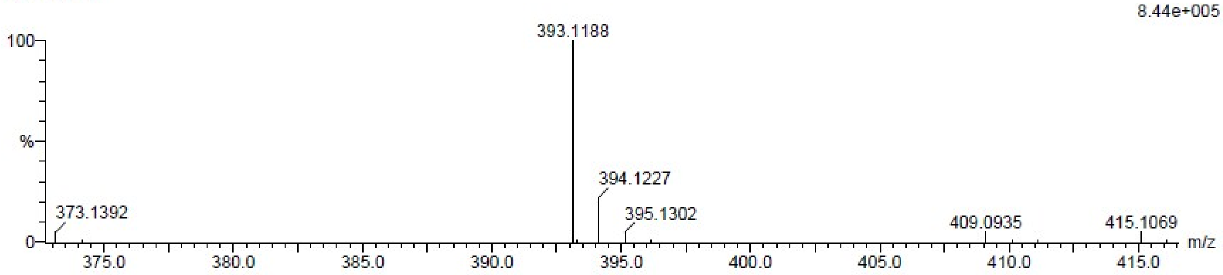

Minimum:				-1.5			
Maximum:		5.0	5.0	100.0			
Mass	Calc. Mass	mDa	PPM	DBE	i-FIT	i-FIT (Norm)	Formula
393.1188	393.1175	1.3	3.3	11.5	28.2	0.0	C18 H18 N4 O5 Na

HRMS spectra of compound **5c**

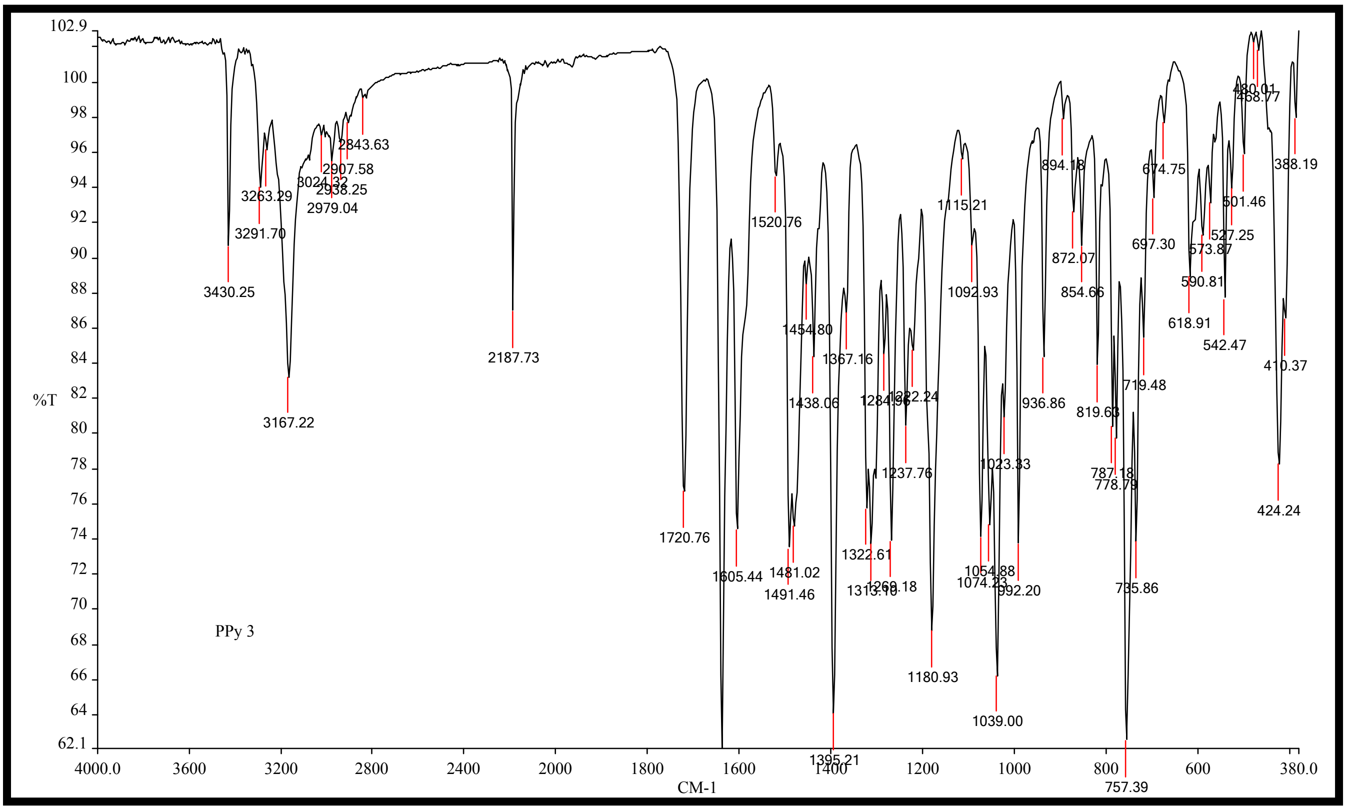

FT-IR spectra of compound **5c**

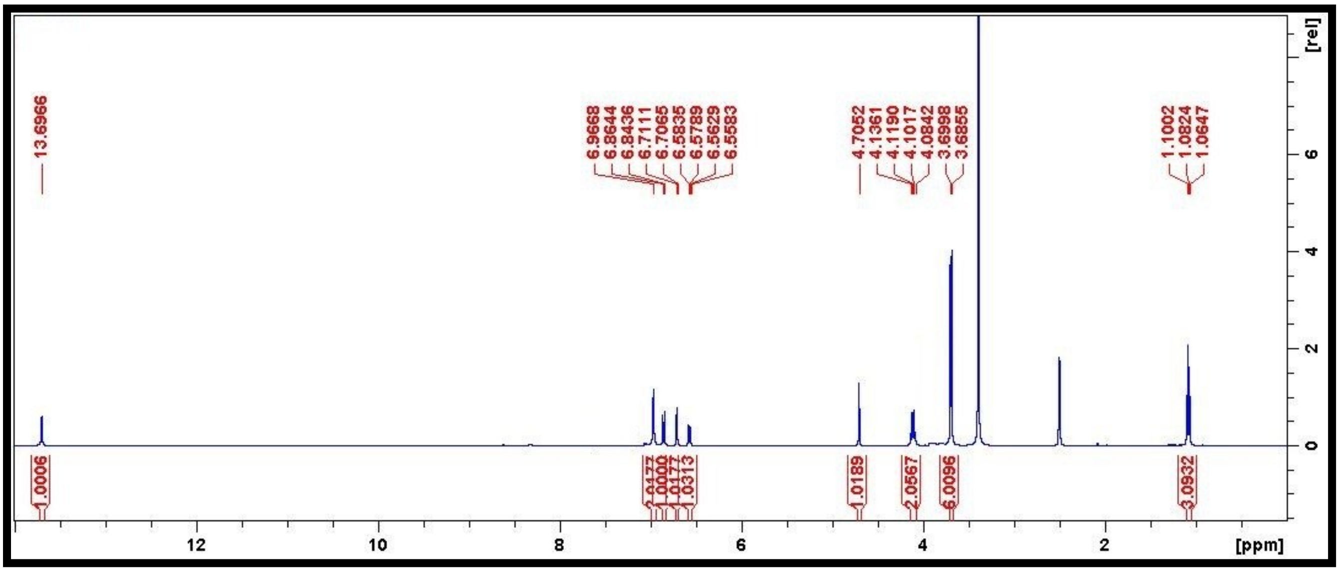

^{1}H NMR spectra of compound **5d**

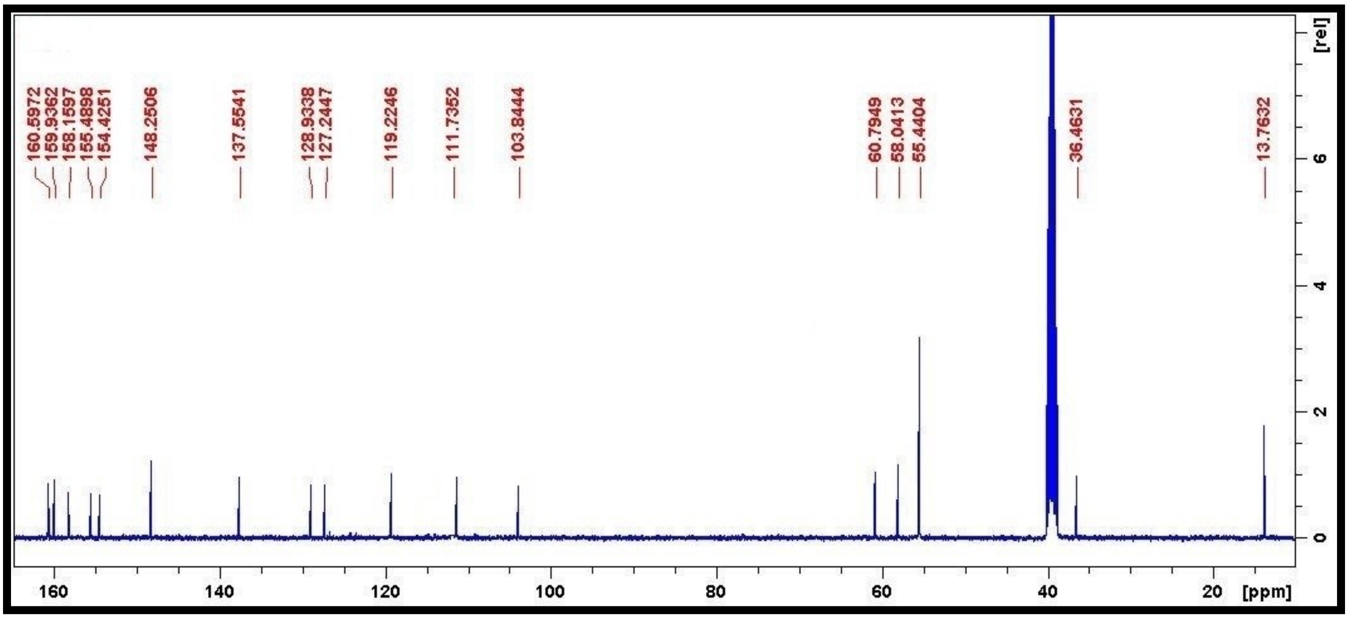

^{13}C NMR spectra of compound **5d**

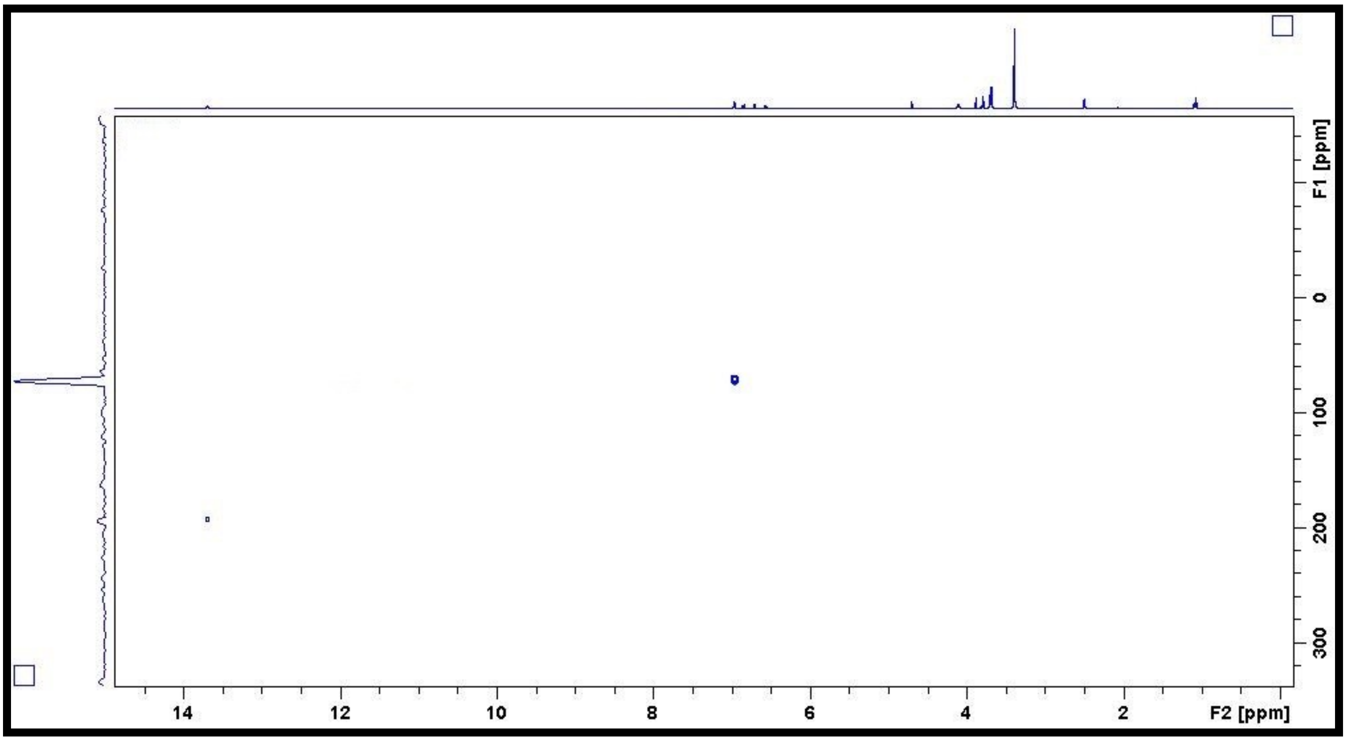

^{15}N NMR spectra of compound **5d**

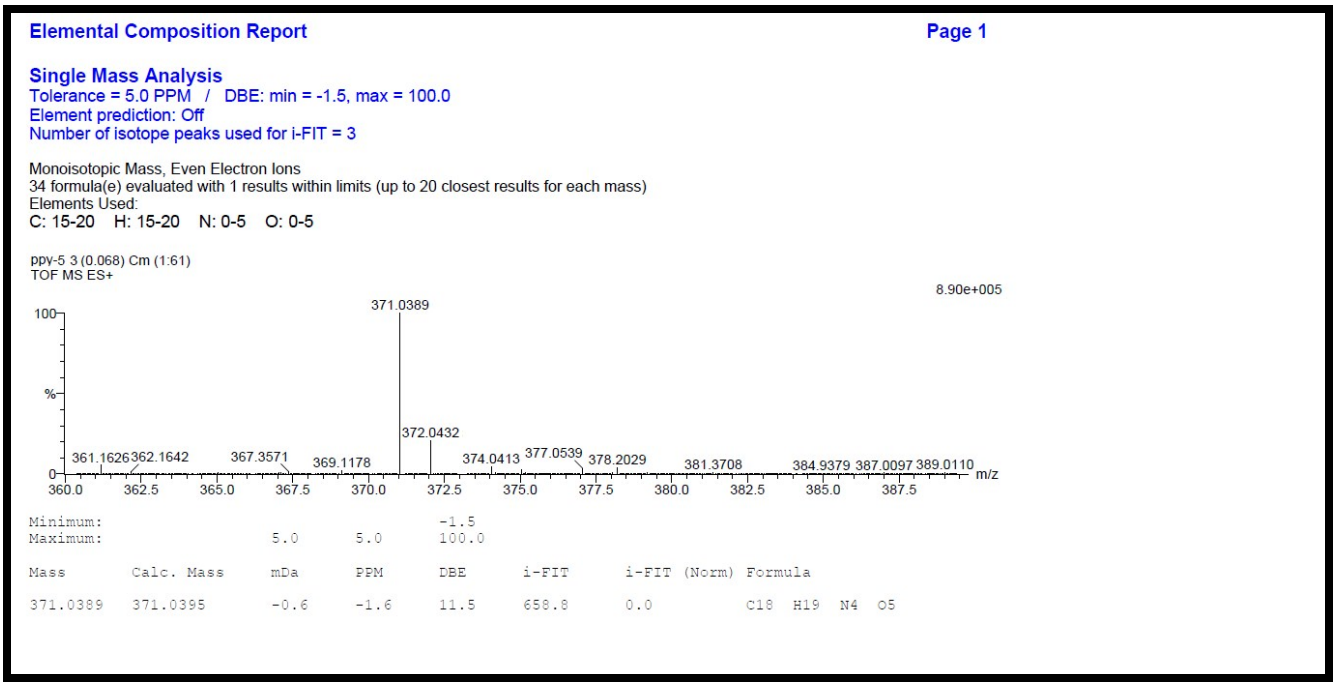

HRMS spectra of compound **5d**

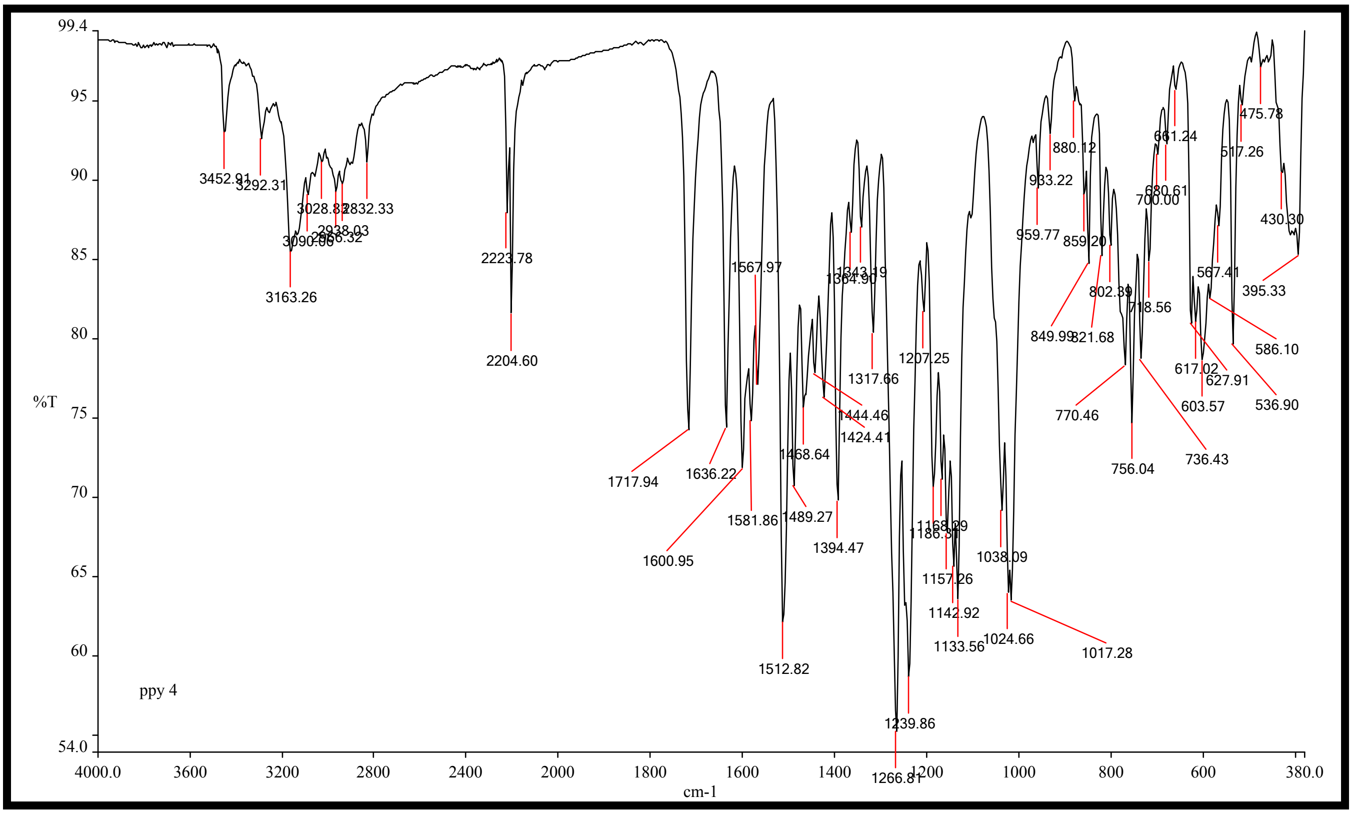

FT-IR spectra of compound **5d**

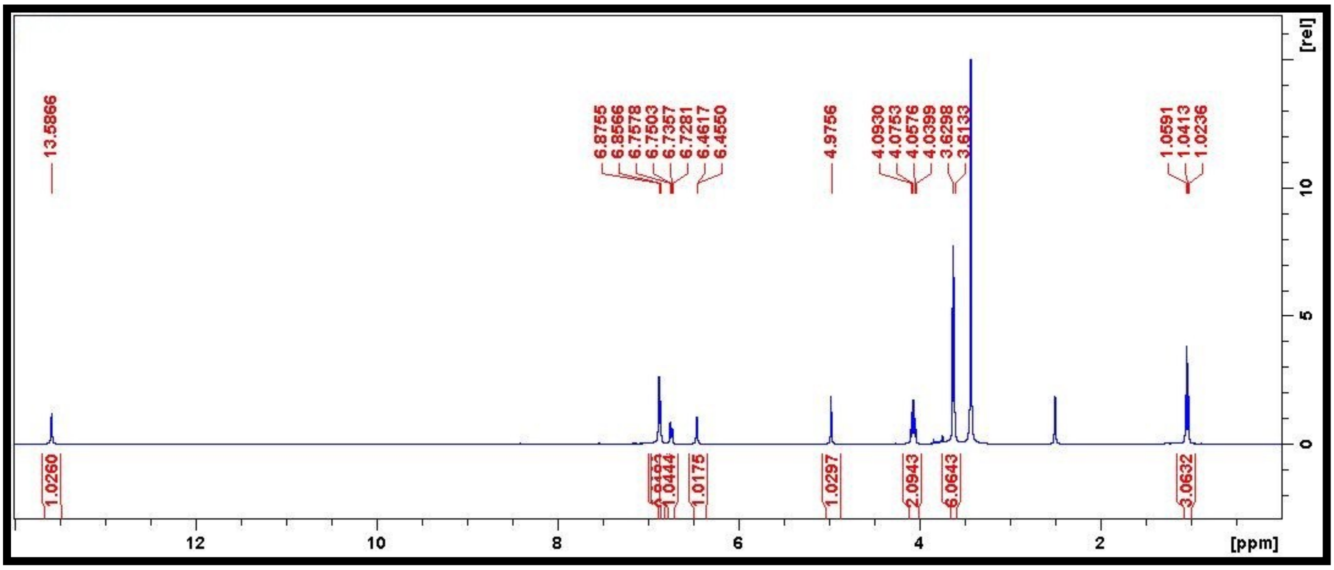

^{1}H NMR spectra of compound **5e**

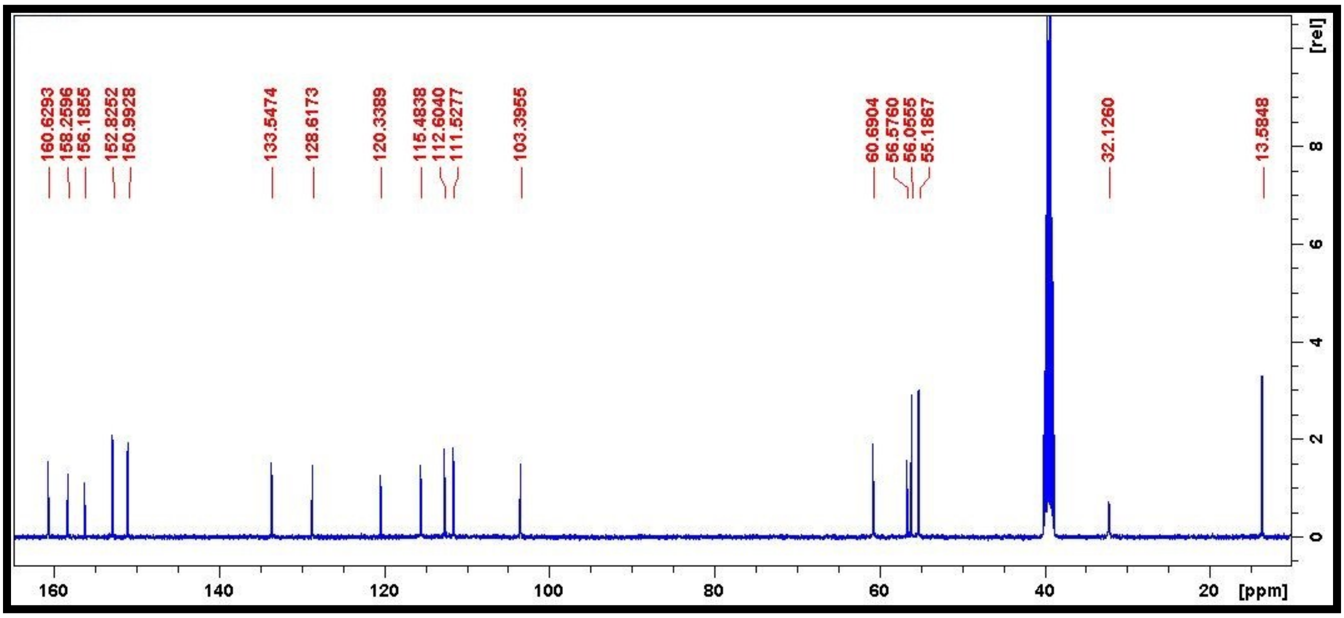

^{13}C NMR spectra of compound **5e**

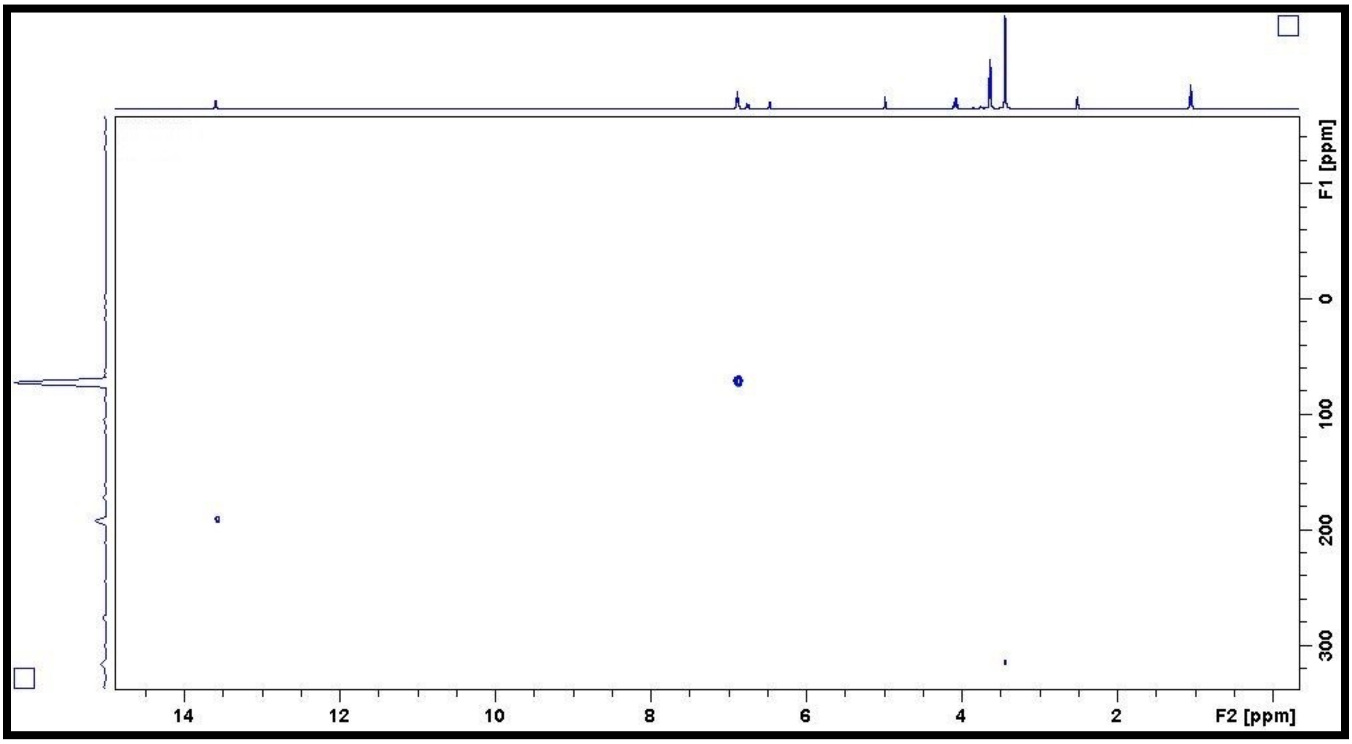

^{15}N NMR spectra of compound **5e**

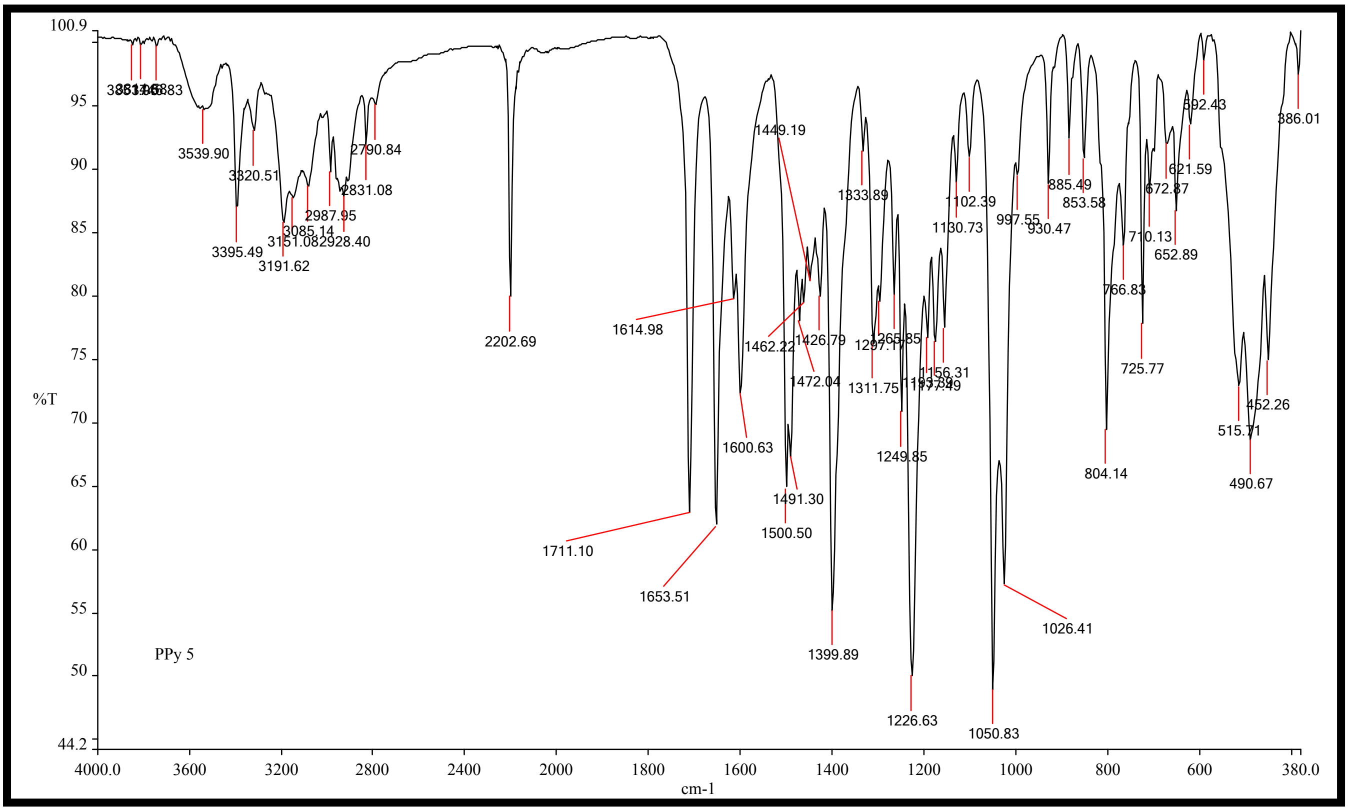

FT-IR spectra of compound **5e**

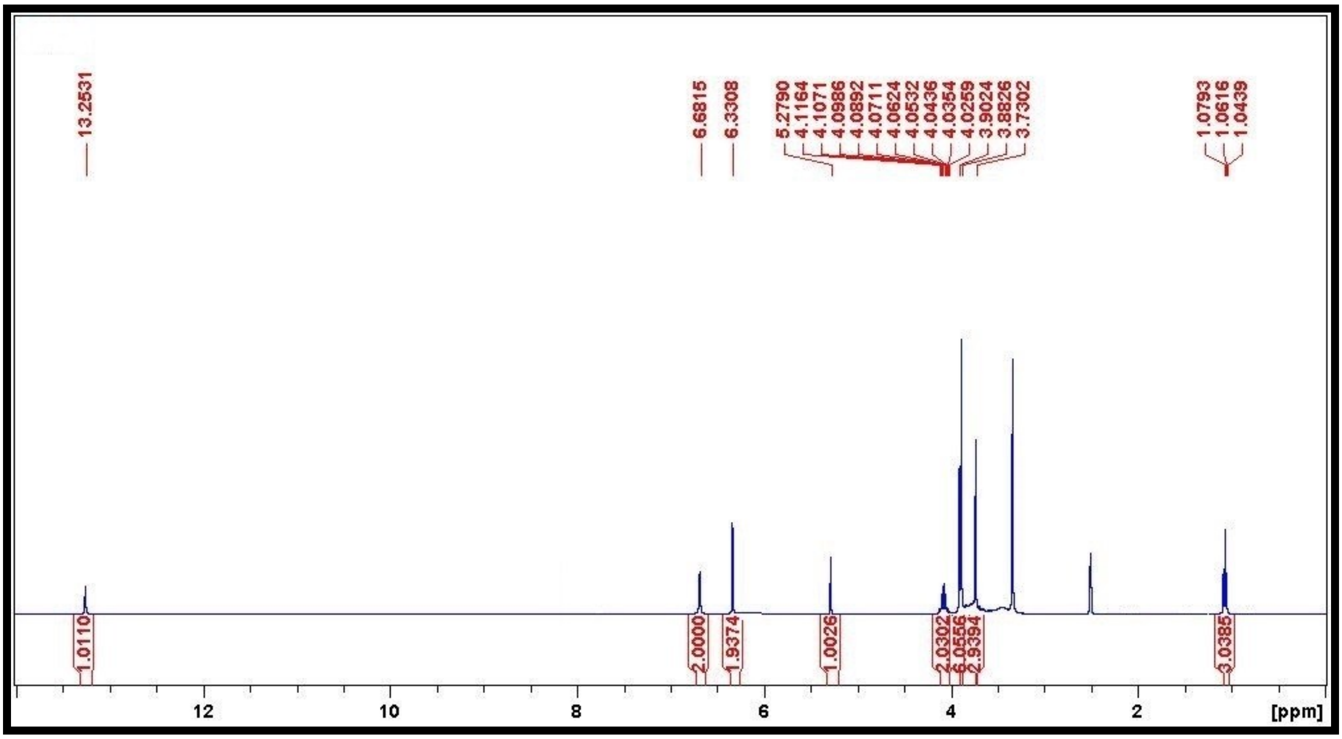

^{1}H NMR spectra of compound **5f**

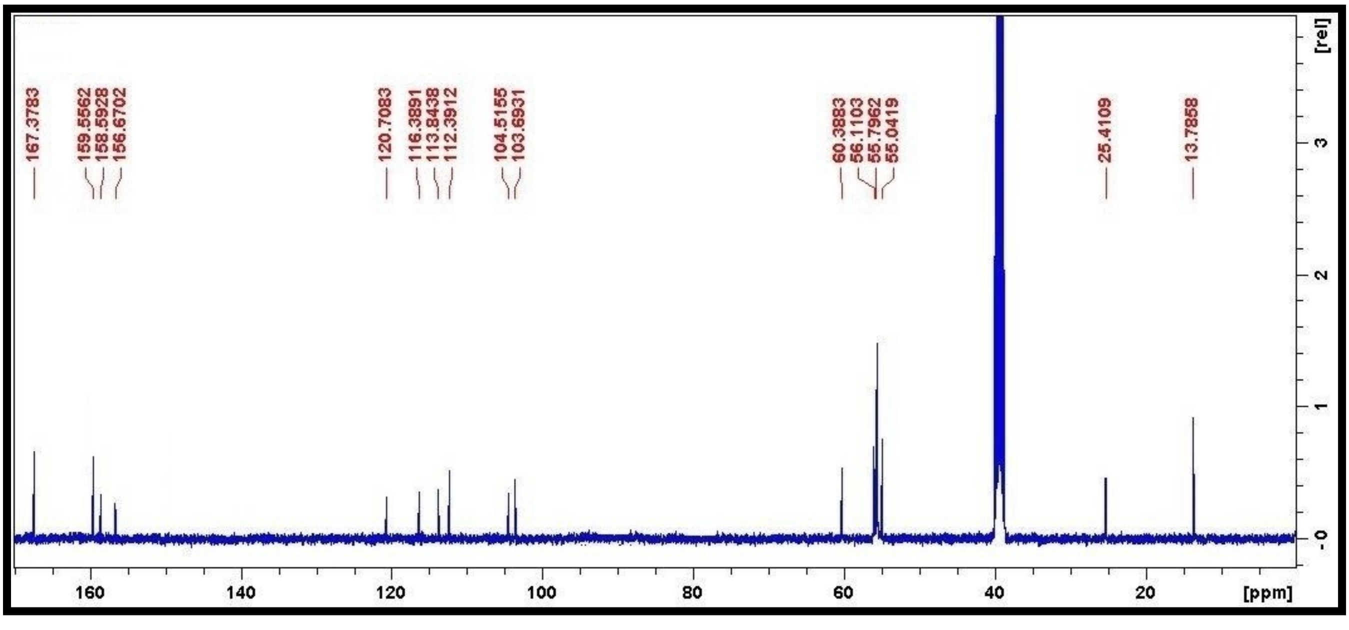

^{13}C NMR spectra of compound **5f**

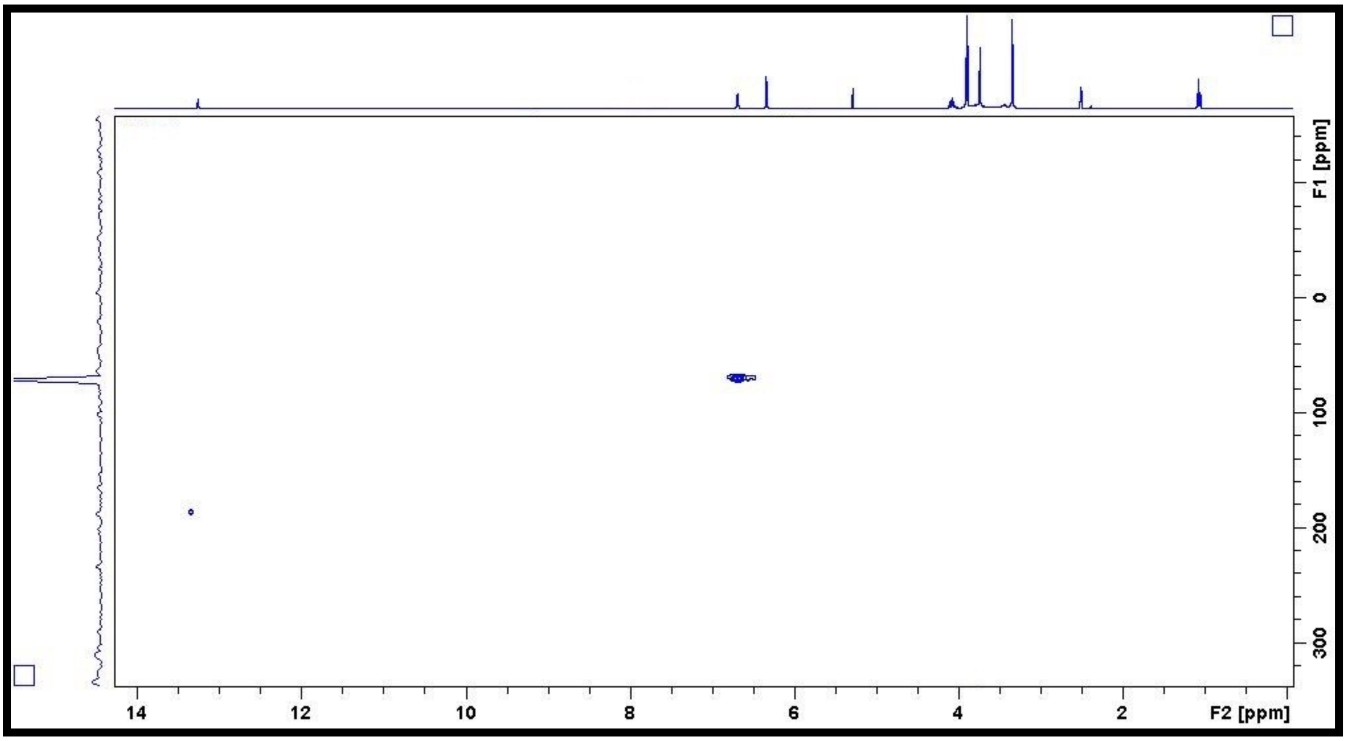

^{15}N NMR spectra of compound **5f**

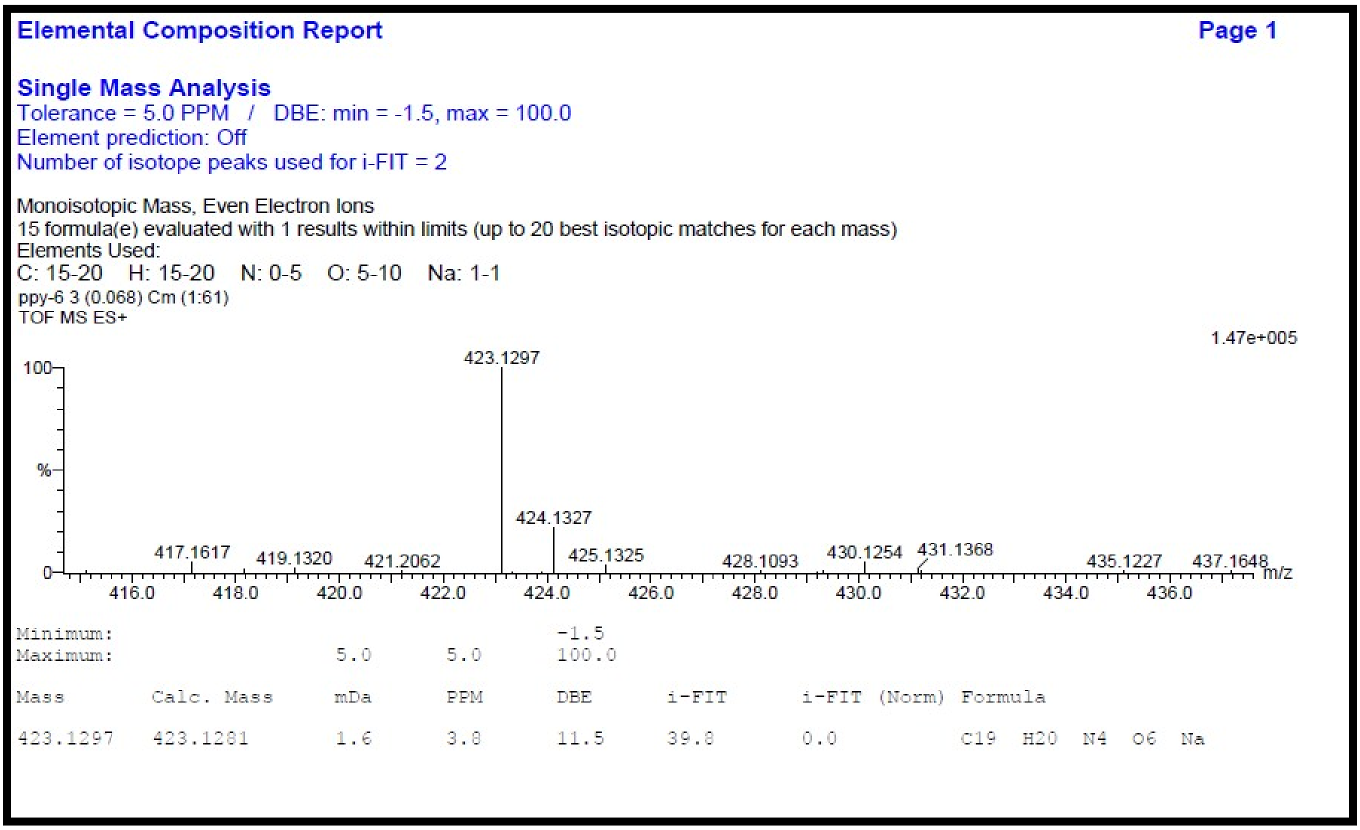

HRMS spectra of compound **5f**

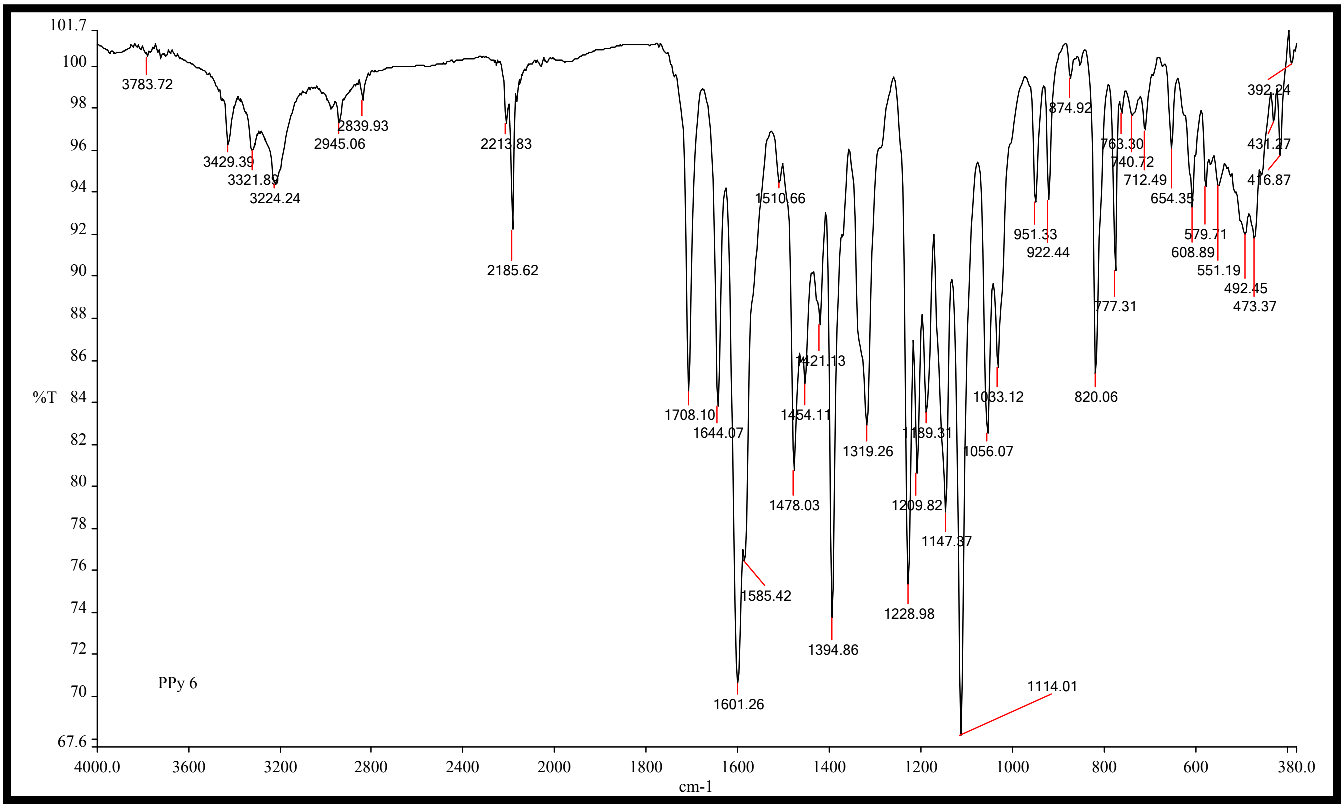

FT-IR spectra of compound **5f**

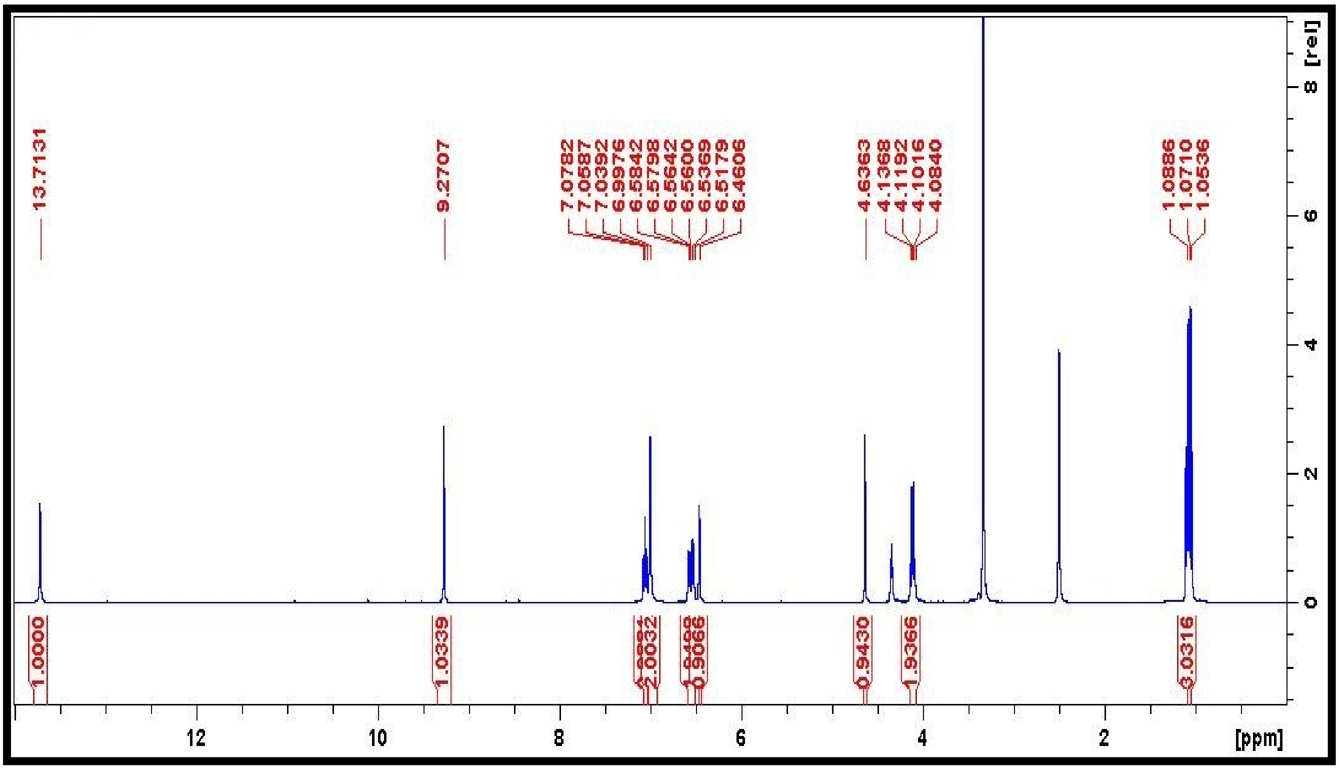

^{1}H NMR spectra of compound **5g**

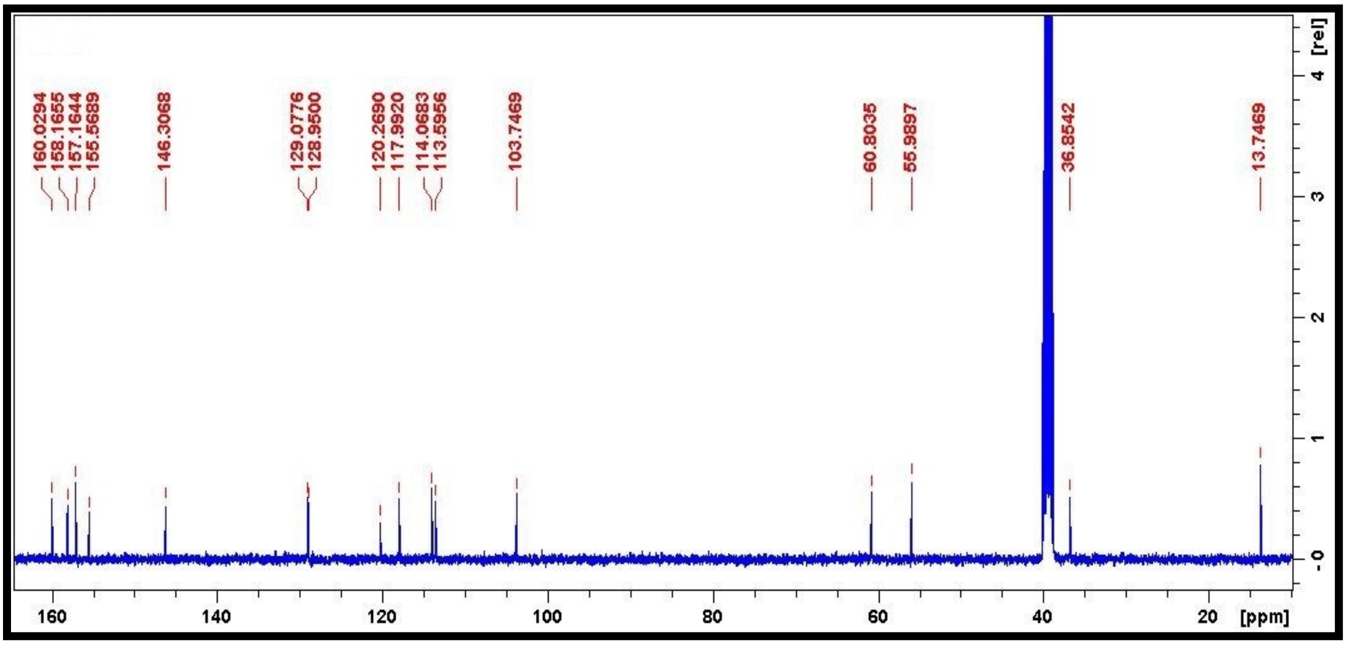

^{13}C NMR spectra of compound **5g**

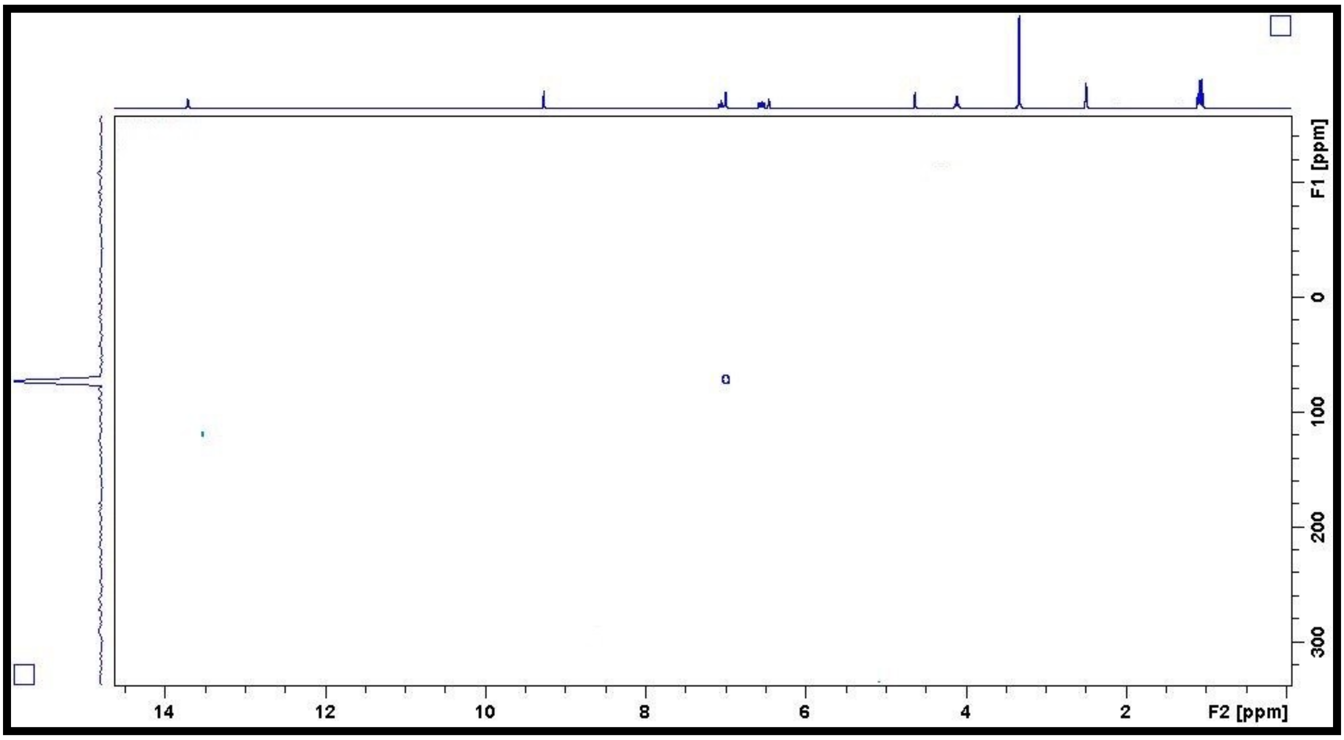

^{15}N NMR spectra of compound **5g**

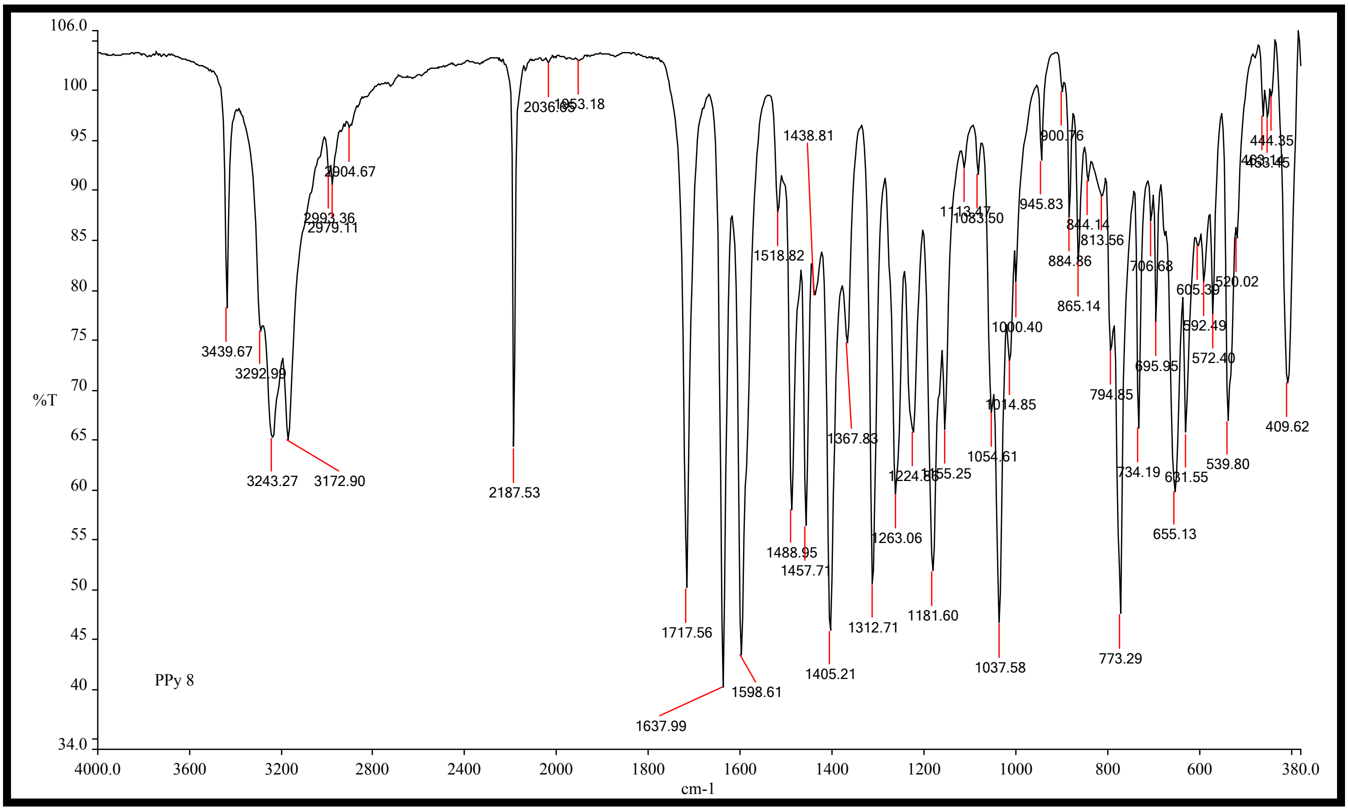

FT-IR spectra of compound **5g**

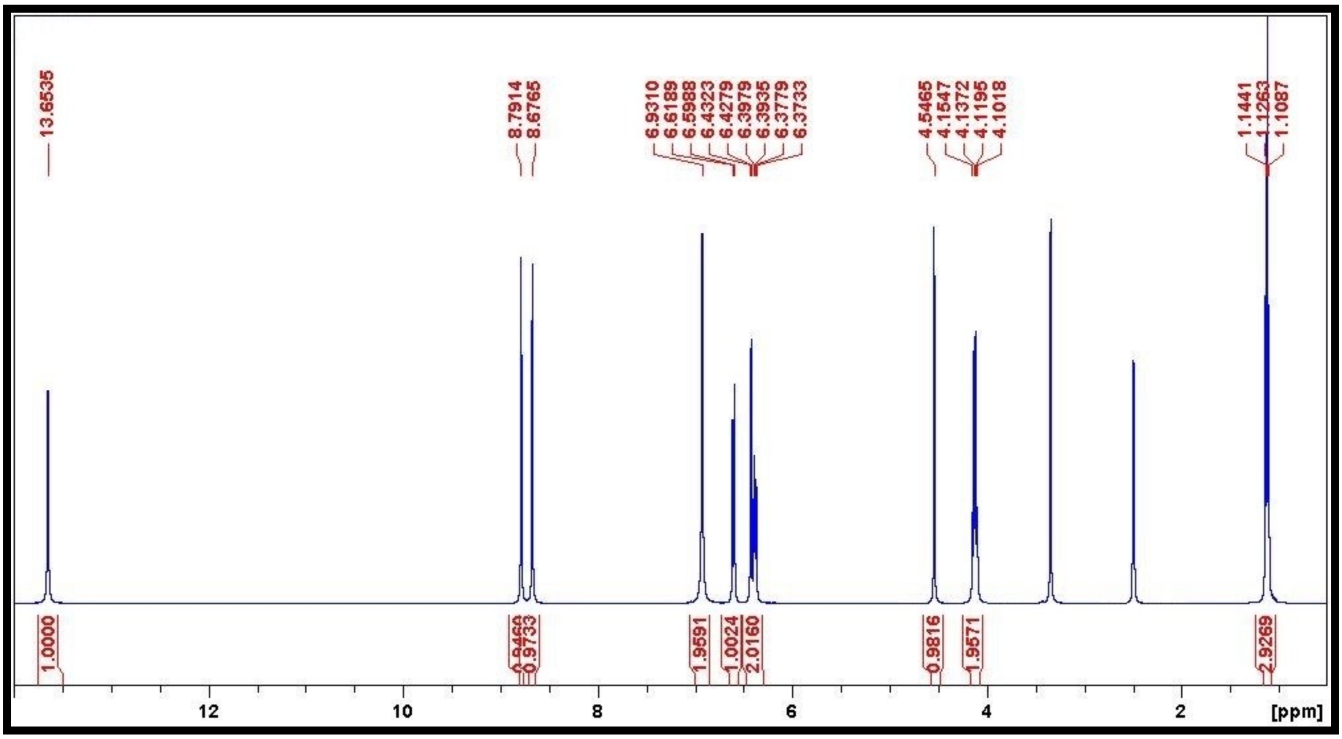

^{1}H NMR spectra of compound **5h**

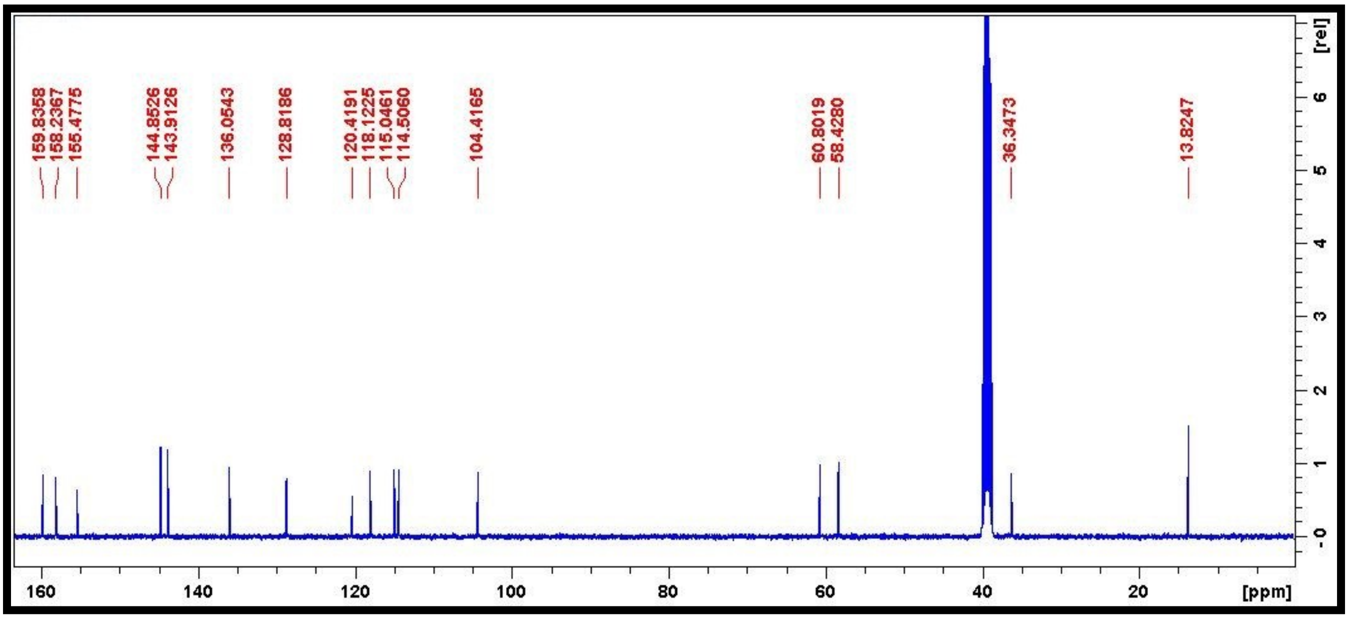

^{13}C NMR spectra of compound **5h**

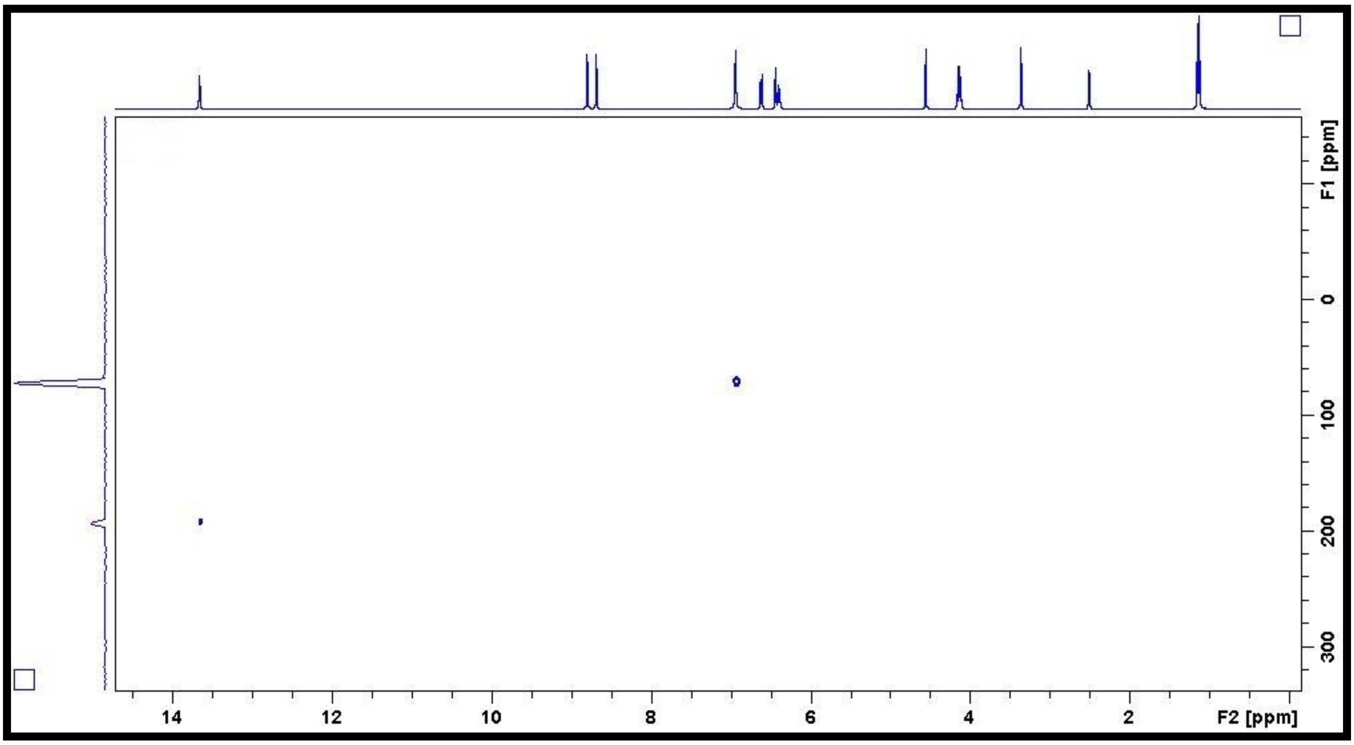

^{15}N NMR spectra of compound **5h**

Elemental Composition Report **Page 1**

Single Mass Analysis
Tolerance = 5.0 PPM / DBE: min = -1.5, max = 100.0
Element prediction: Off
Number of isotope peaks used for i-FIT = 2

Monoisotopic Mass, Even Electron Ions
15 formula(e) evaluated with 1 results within limits (up to 20 best isotopic matches for each mass)
Elements Used:
C: 15-20 H: 10-15 N: 0-5 O: 0-5
ppy-9 61 (2.024) Cm (1:61)
TOF MS ES-

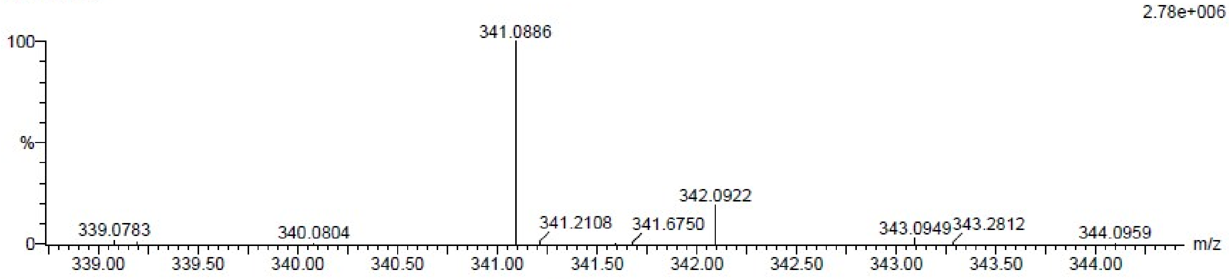

Minimum:				-1.5			
Maximum:		5.0	5.0	100.0			
Mass	Calc. Mass	mDa	PPM	DBE	i-FIT	i-FIT (Norm)	Formula
341.0886	341.0886	0.0	0.0	12.5	74.2	0.0	C16 H13 N4 O5

HRMS spectra of compound **5h**

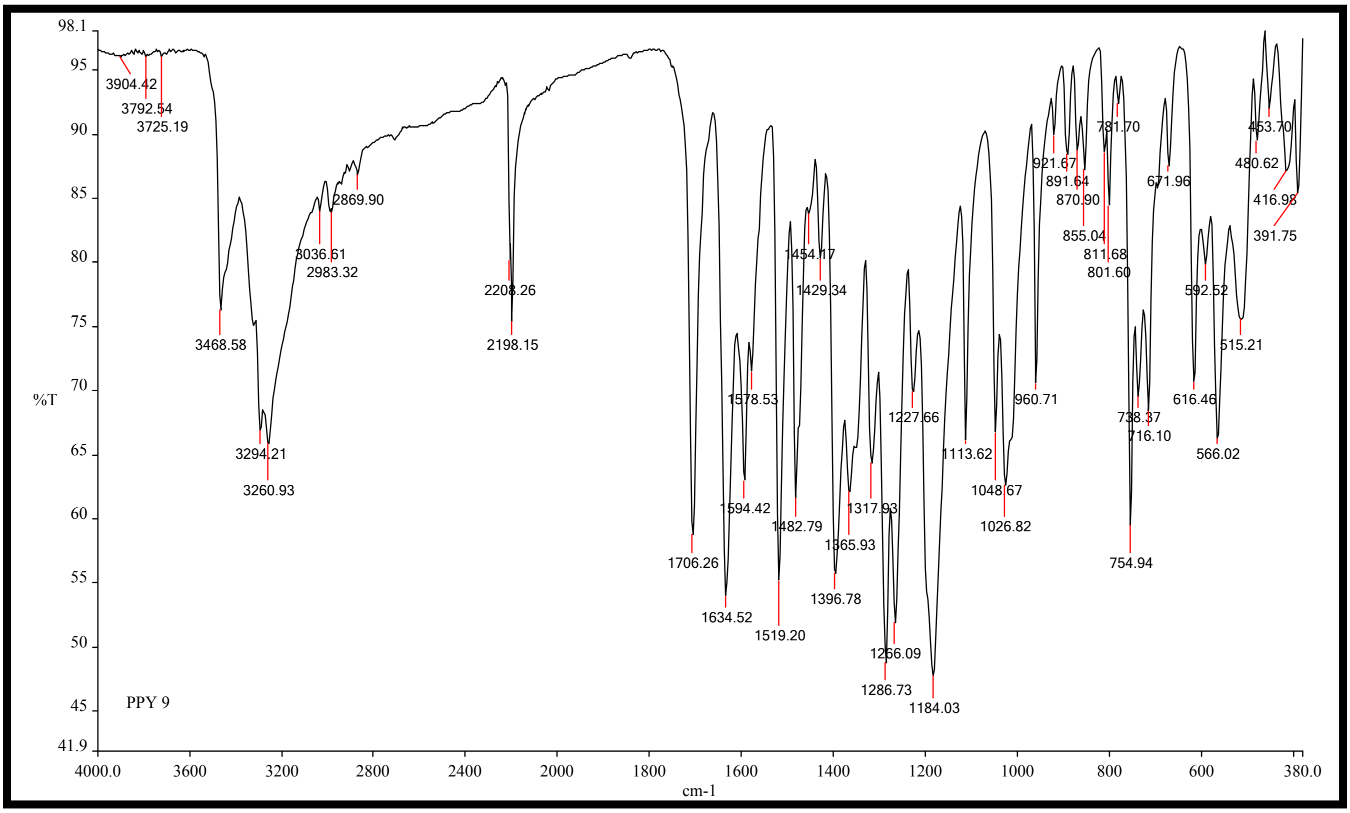

FT-IR spectra of compound **5h**

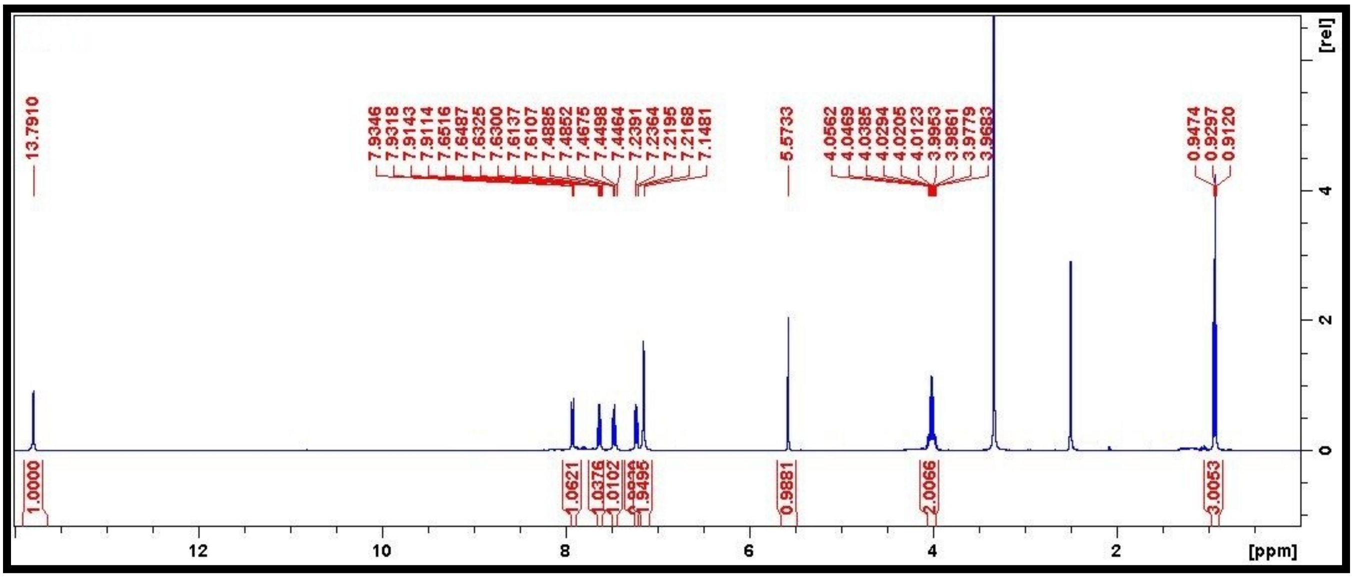

^{1}H NMR spectra of compound **5i**

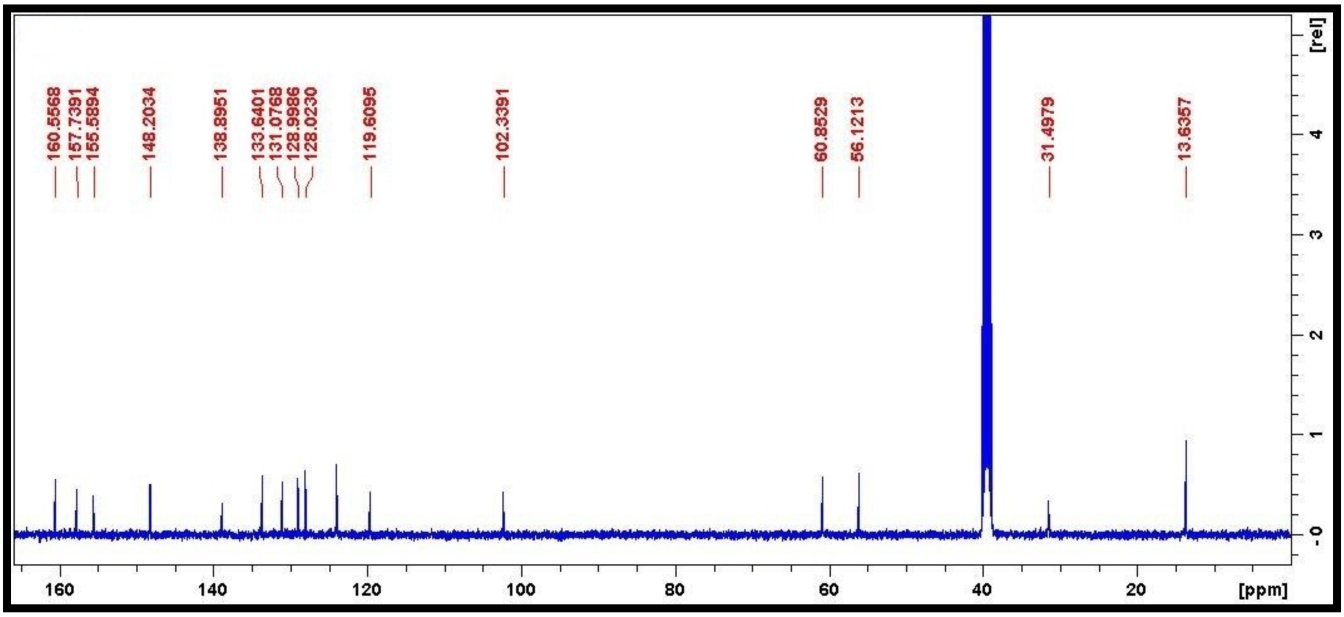

^{13}C NMR spectra of compound **5i**

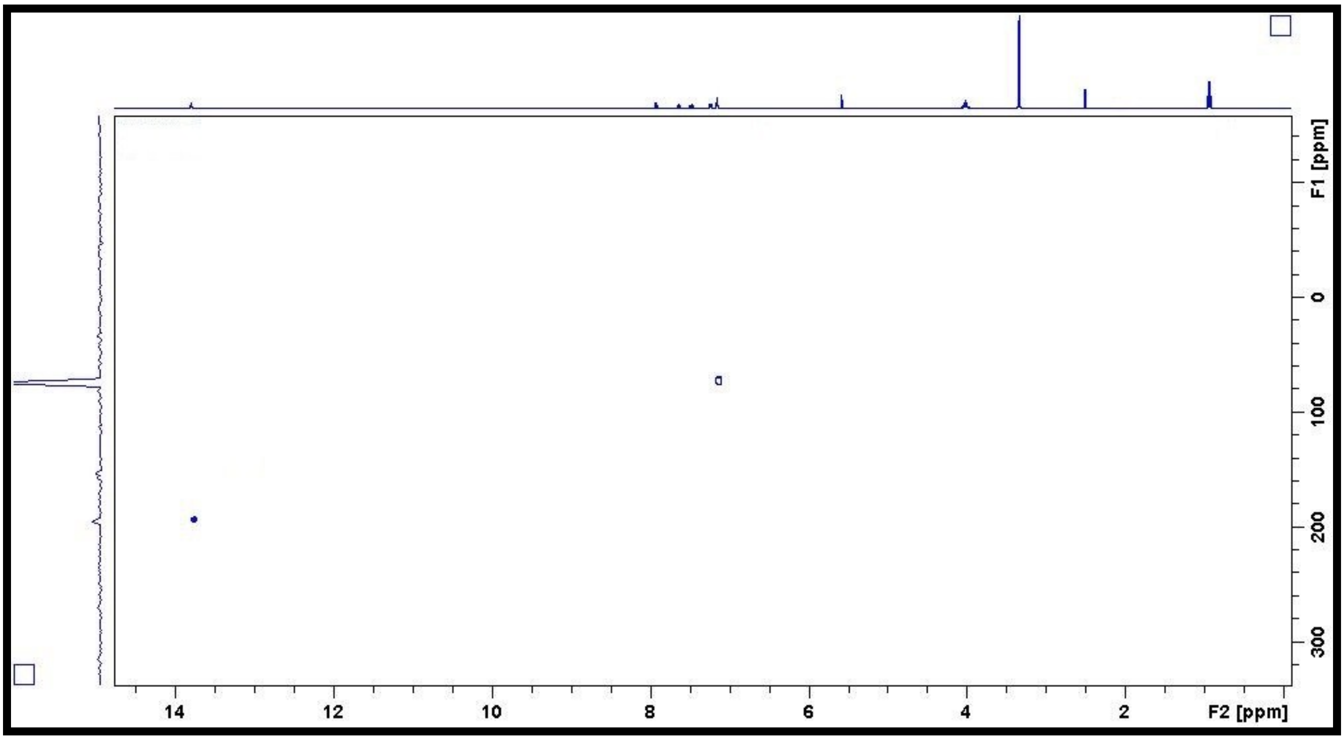

^{15}N NMR spectra of compound **5i**

Elemental Composition Report | **Page 1**

Single Mass Analysis
Tolerance = 5.0 PPM / DBE: min = -1.5, max = 100.0
Element prediction: Off
Number of isotope peaks used for i-FIT = 2

Monoisotopic Mass, Even Electron Ions
15 formula(e) evaluated with 1 results within limits (up to 20 best isotopic matches for each mass)
Elements Used:
C: 15-20 H: 10-15 N: 0-5 O: 0-5
ppy-11 6 (0.169) Cm (1:61)
TOF MS ES-

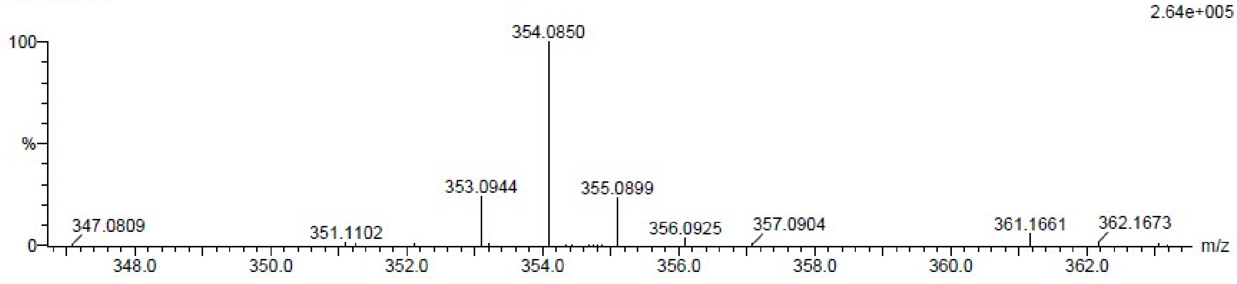

Minimum:			-1.5		
Maximum:	5.0	5.0	100.0		

Mass	Calc. Mass	mDa	PPM	DBE	i-FIT	i-FIT (Norm)	Formula
354.0850	354.0838	1.2	3.4	13.5	103.8	0.0	C16 H12 N5 O5

HRMS spectra of compound **5i**

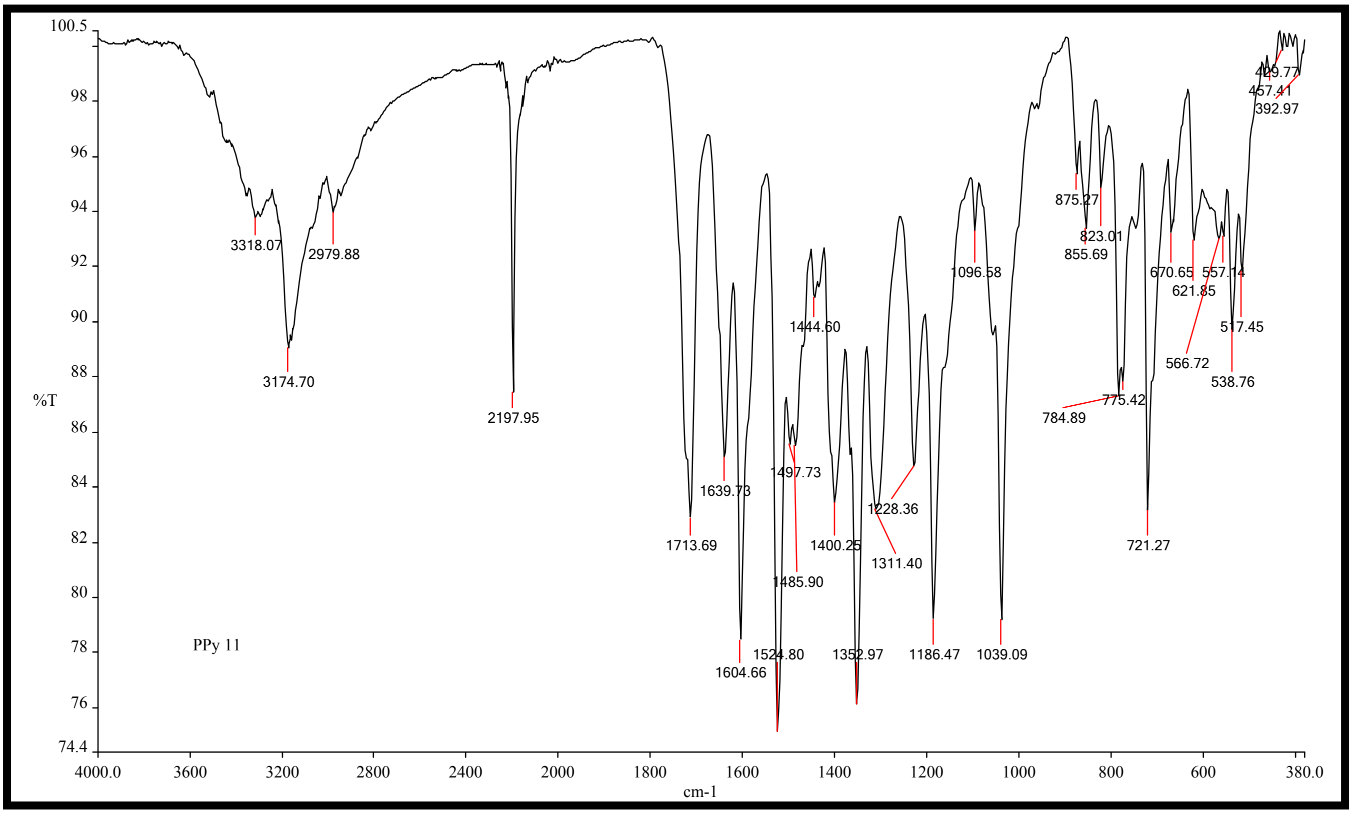

FT-IR spectra of compound **5i**

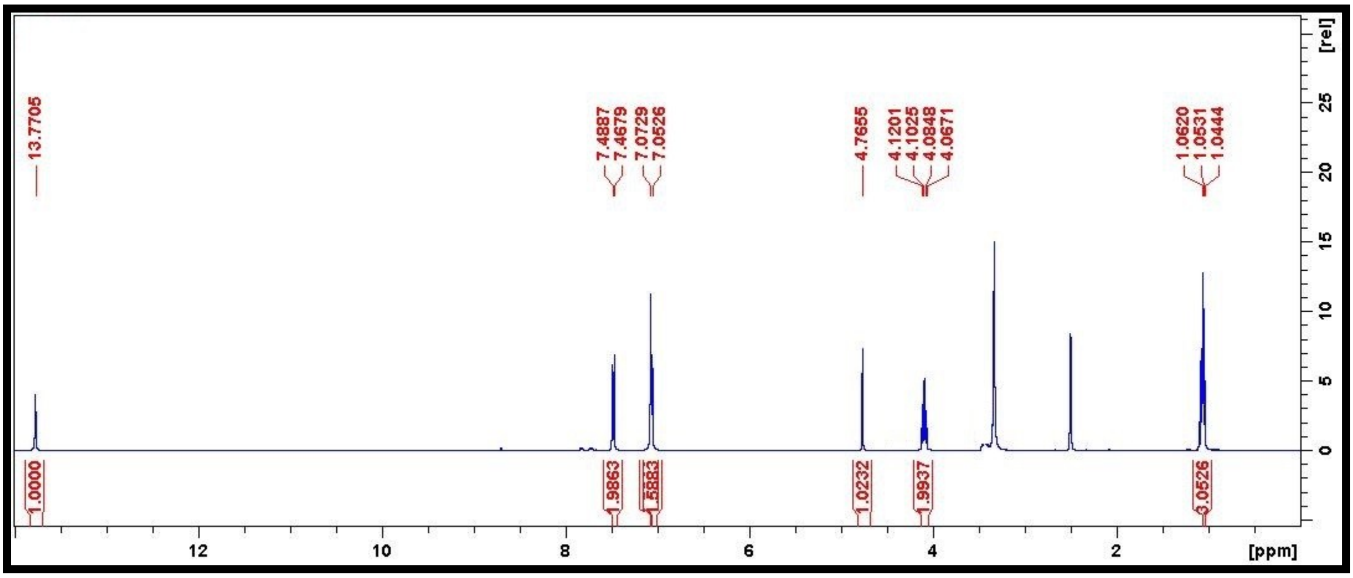

^{1}H NMR spectra of compound **5j**

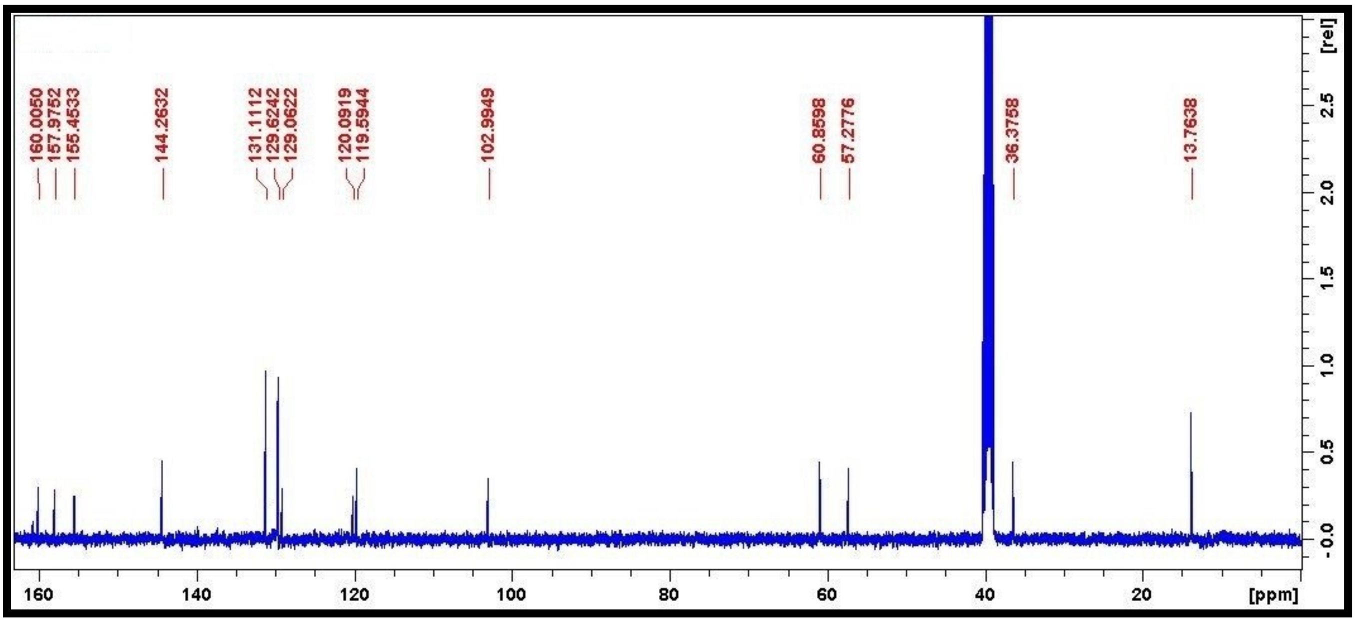

^{13}C NMR spectra of compound **5j**

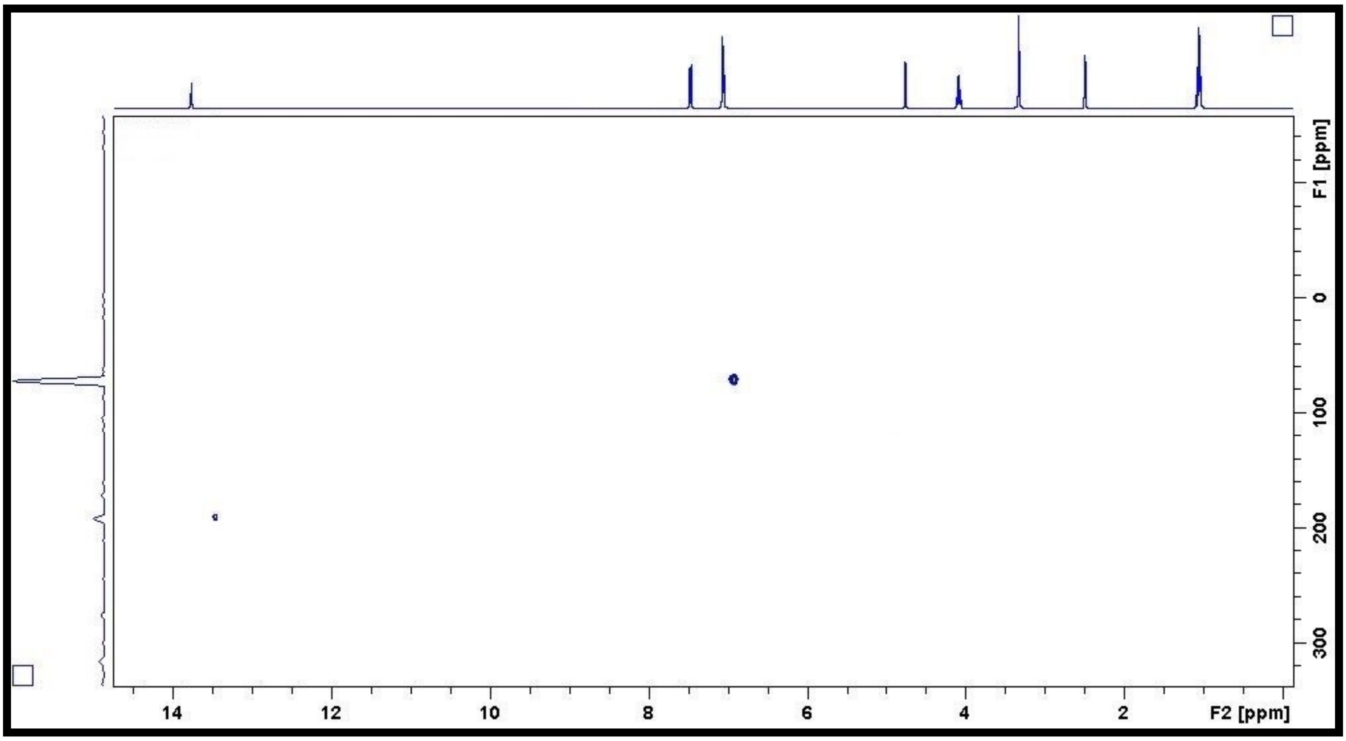

^{15}N NMR spectra of compound **5j**

Elemental Composition Report Page 1

Single Mass Analysis
Tolerance = 5.0 PPM / DBE: min = -1.5, max = 100.0
Element prediction: Off
Number of isotope peaks used for i-FIT = 2

Monoisotopic Mass, Even Electron Ions
40 formula(e) evaluated with 1 results within limits (up to 20 best isotopic matches for each mass)
Elements Used:
C: 15-20 H: 10-15 N: 0-5 O: 0-5 Br: 0-1

ppy-13 41 (1.349) Cm (1:61)
TOF MS ES-

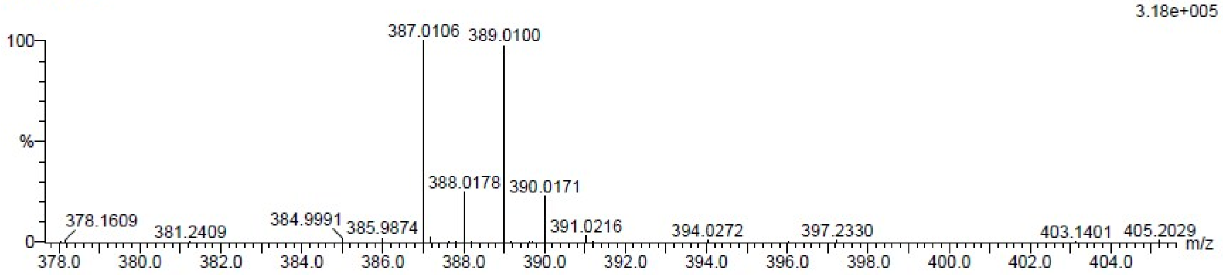

Minimum:				-1.5			
Maximum:		5.0	5.0	100.0			
Mass	Calc. Mass	mDa	PPM	DBE	i-FIT	i-FIT (Norm)	Formula
387.0106	387.0093	1.3	3.4	12.5	85.4	0.0	C16 H12 N4 O3 Br

HRMS spectra of compound **5j**

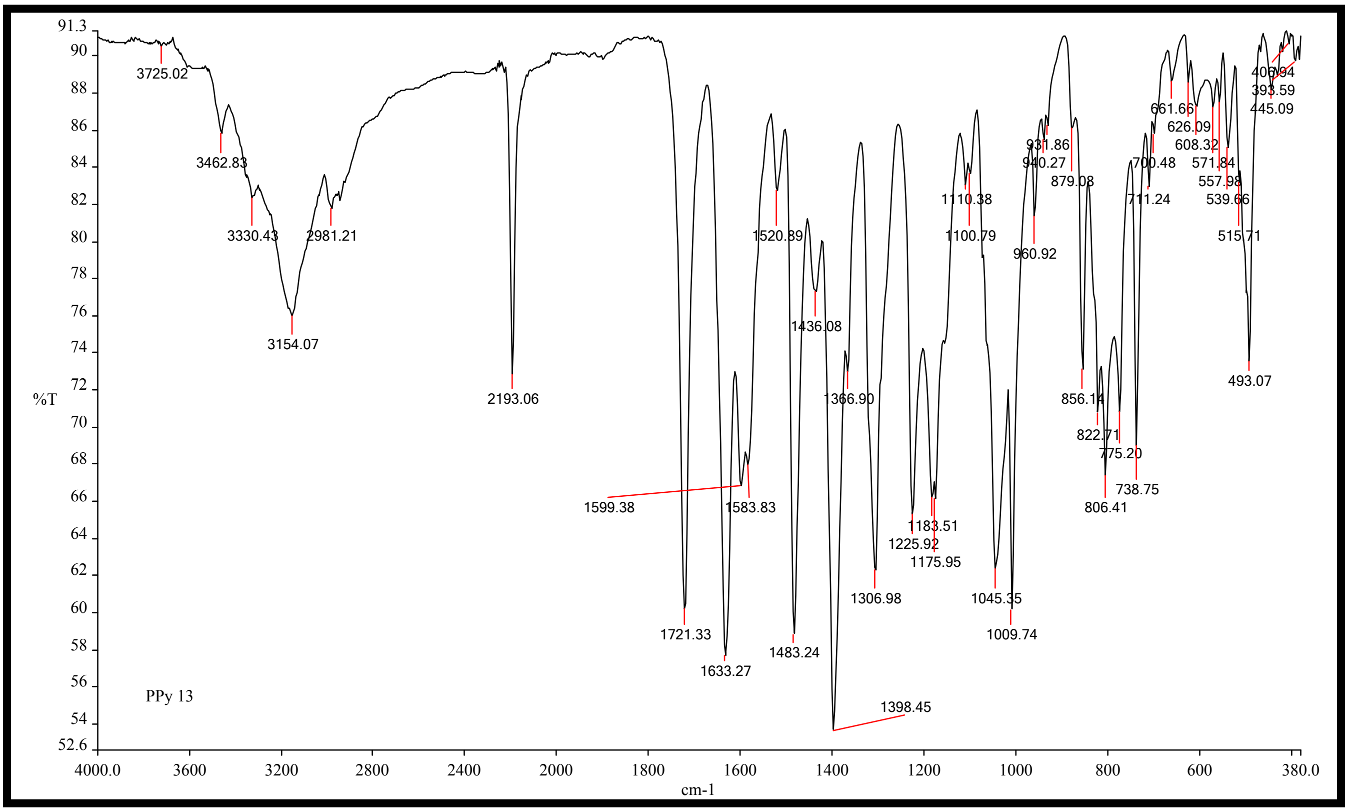

^{1}H NMR spectra of compound **5j**

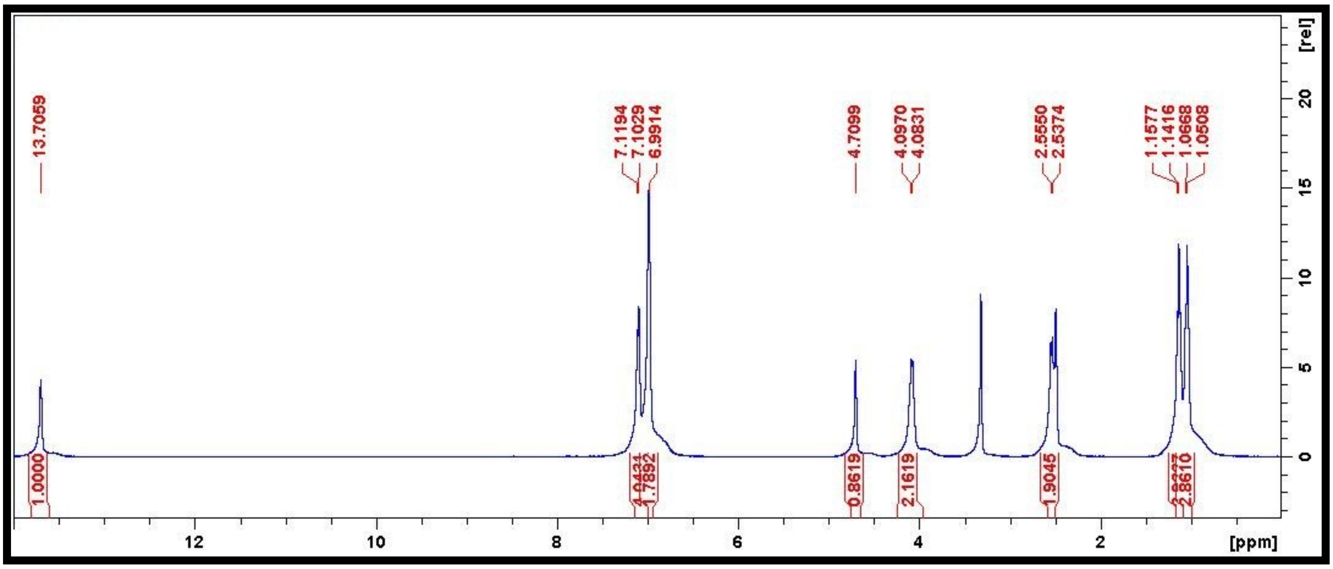

^{1}H NMR spectra of compound **5k**

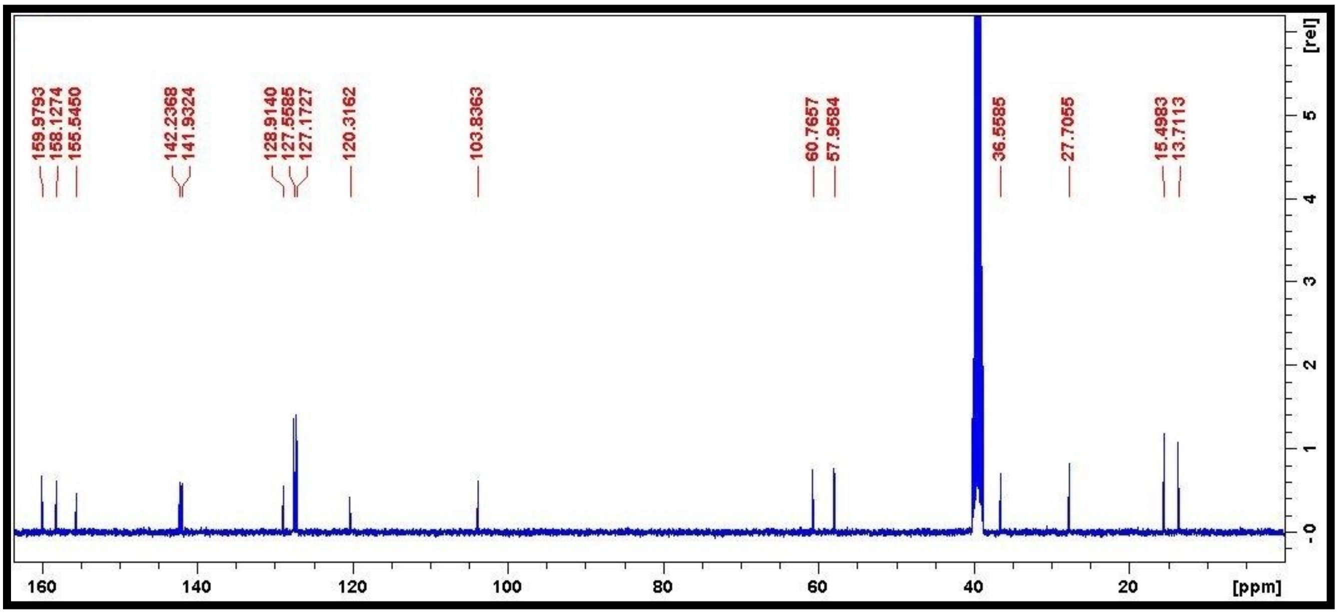

^{13}C NMR spectra of compound **5k**

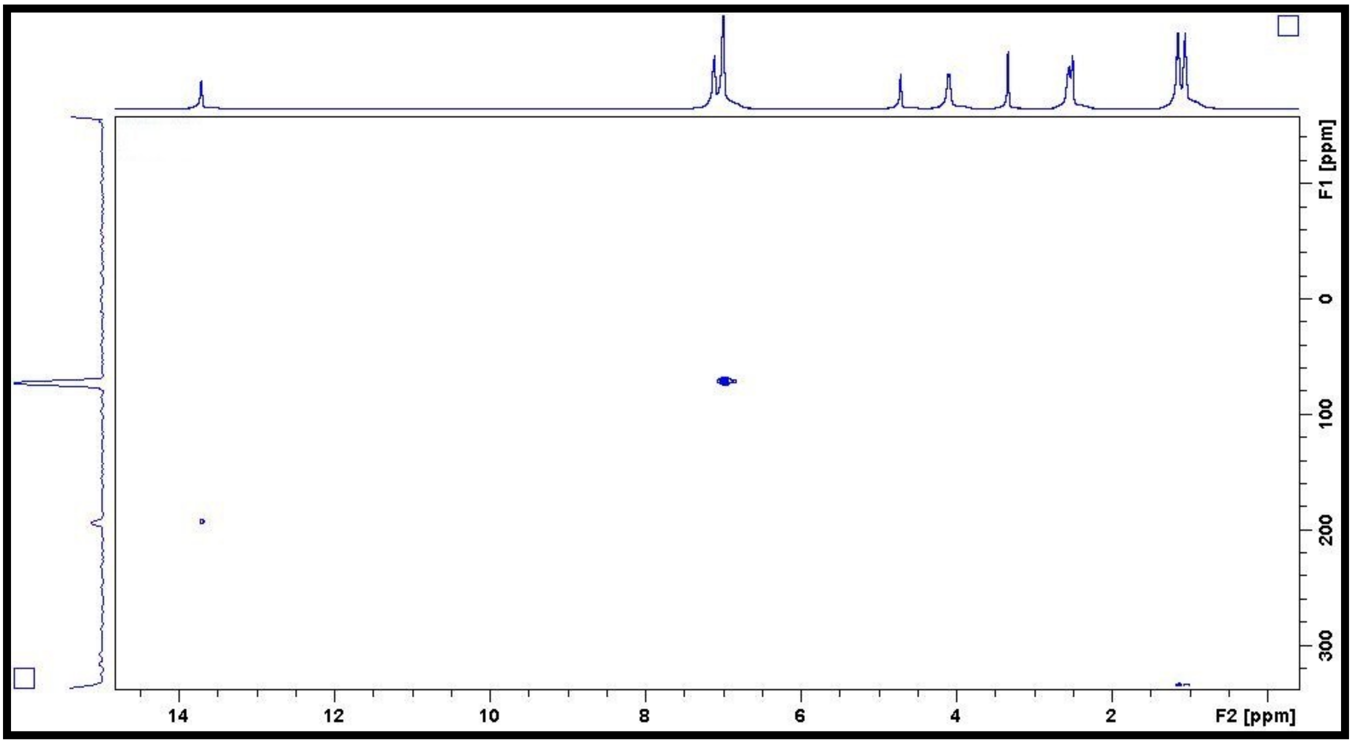

^{15}N NMR spectra of compound **5k**

Elemental Composition Report | **Page 1**

Single Mass Analysis
Tolerance = 5.0 PPM / DBE: min = -1.5, max = 100.0
Element prediction: Off
Number of isotope peaks used for i-FIT = 2

Monoisotopic Mass, Even Electron Ions
15 formula(e) evaluated with 1 results within limits (up to 20 best isotopic matches for each mass)
Elements Used:
C: 15-20 H: 15-20 N: 0-5 O: 0-5
ppy-14 24 (0.776) Cm (1:61)
TOF MS ES-

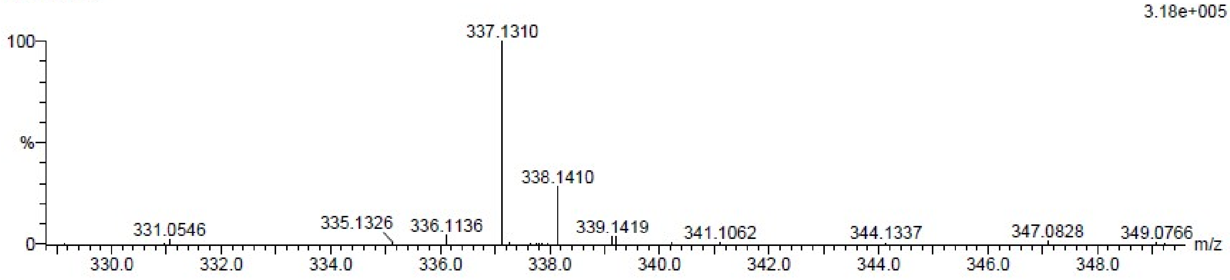

Minimum:				-1.5			
Maximum:		5.0	5.0	100.0			
Mass	Calc. Mass	mDa	PPM	DBE	i-FIT	i-FIT (Norm)	Formula
337.1310	337.1301	0.9	2.7	12.5	112.8	0.0	C18 H17 N4 O3

HRMS spectra of compound **5k**

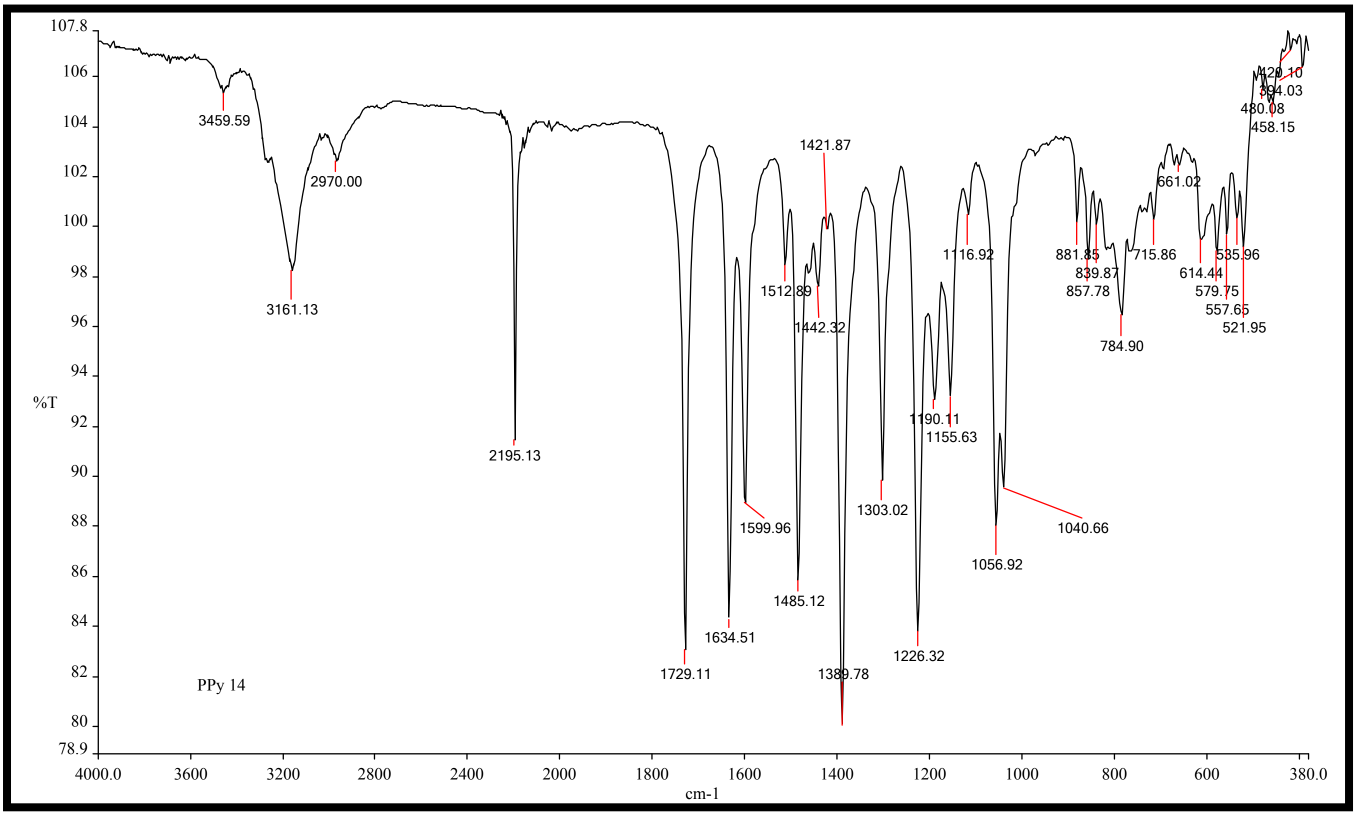

FT-IR spectra of compound **5k**
